# Podocytes

**DOI:** 10.12688/f1000research.7255.1

**Published:** 2016-01-28

**Authors:** Jochen Reiser, Mehmet M. Altintas

**Affiliations:** 1 Department of Medicine, Rush University Medical Center, Chicago, IL, USA

**Keywords:** Podocytes, kidney glomerulus, urinary ultrafiltrate, glomerular filtration

## Abstract

Podocytes are highly specialized cells of the kidney glomerulus that wrap around capillaries and that neighbor cells of the Bowman’s capsule. When it comes to glomerular filtration, podocytes play an active role in preventing plasma proteins from entering the urinary ultrafiltrate by providing a barrier comprising filtration slits between foot processes, which in aggregate represent a dynamic network of cellular extensions. Foot processes interdigitate with foot processes from adjacent podocytes and form a network of narrow and rather uniform gaps. The fenestrated endothelial cells retain blood cells but permit passage of small solutes and an overlying basement membrane less permeable to macromolecules, in particular to albumin. The cytoskeletal dynamics and structural plasticity of podocytes as well as the signaling between each of these distinct layers are essential for an efficient glomerular filtration and thus for proper renal function. The genetic or acquired impairment of podocytes may lead to foot process effacement (podocyte fusion or retraction), a morphological hallmark of proteinuric renal diseases. Here, we briefly discuss aspects of a contemporary view of podocytes in glomerular filtration, the patterns of structural changes in podocytes associated with common glomerular diseases, and the current state of basic and clinical research.

## Podocytes and glomerular filtration

Podocytes (or visceral epithelial cells) are terminally differentiated cells lining the outer surface of the glomerular capillaries. As a major component of the ultrafiltration apparatus, podocytes have a complex cellular architecture consisting of cell body, major processes that extend outward from their cell body, forming interdigitated foot processes (FPs) that enwrap the glomerular capillaries ^1^. Major processes are tethered by microtubules and intermediate filaments while FPs contain actin-based cytoskeleton ^2– 4^. Podocyte FPs comprise a functioning slit diaphragm (SD) in between ^5, 6^, a meshwork of proteins actively participating in podocyte signaling ^7– 9^. In addition, FPs have a thick, negatively charged coat (glycocalyx) facing the urinary space ^10^; this accounts for negative surface charges throughout the glomerular filtration barrier, which generates an electrostatic repel between the neighboring FPs and helps maintain the unique cytoarchitecture of podocytes by enhancing the physical separation ^11^. Podocytes form the glomerular filtration barrier together with the opposing monolayers of fenestrated endothelium in the vascular space ^12^ and glomerular basement membrane (GBM) in between ^13, 14^. This three-layer filtration barrier serves as a size-selective and charge-dependent molecular sieve facilitating the filtration of cationic molecules, electrolytes, and small and midsized solutes but restricting the passage of anionic molecules and macromolecules ^15, 16^. It is important to bear in mind that those layers should be arranged with decreasing selectivity, with the SD being the least selective filter; otherwise, retained plasma proteins would routinely accumulate behind the filtration slits of podocytes ^17^. This elegant structure has to oppose hydrostatic pressure in the glomerular capillary, which is the natural driving force behind macromolecular filtration.

If podocytes are injured, mutated, or lost, the elaborate structure of podocytes is physically altered—a process termed ‘foot process effacement’, which is found in many proteinuric kidney diseases. In some cases, once FPs are effaced (flattened down and fused), the glomerular filtration barrier is no longer intact as evidently indicated by the massive leak of proteins out of the vasculature into the urine, known as proteinuria ^18^. Proteinuria (also referred to as ‘albuminuria’ or ‘microalbuminuria’) is a clinically important sign of early renal dysfunction. In the following sections, we outline the response of podocytes to various stimuli or injury (or both) to better understand the mechanisms underlying podocyte FP effacement, proteinuria, and glomerular disease progression.

## Major causes of podocyte injury

Podocyte function depends on a highly ordered cellular arrangement of filtration compartments and the correct signaling within this microenvironment. Therefore, podocytes are uniquely sensitive to a variety of agents interfering with their actin cytoskeleton, their apical membrane domain (i.e., the negative surface charge), SD complex that regulates podocyte actin reorganization, and GBM structure to which podocytes adhere ^19– 21^. The mechanisms leading to podocytopathies at the molecular level include genetic events (genetic mutations and deletions) associated with common complex diseases ^22, 23^.

The core structural component of podocyte FPs is a highly regulated actin cytoskeletal network, which was represented either by a dense bundle of actin filaments that extends along the length of FPs or by a relatively short and branched cortical network, which is located at the cell periphery and anchors elements of the SD. The initial response of podocytes to injury is the disruption of these structures and actin dysregulation, where actin and actin-binding proteins accumulate.

In experimental models aiming to study various aspects of cell and molecular biology of podocytes, it has been demonstrated that podocytes are the major targets of various soluble and cellular products, including toxins, reactive oxygen species (ROS), complements, and antibodies, as outlined in Table 1.

**Table 1.** Agents, molecules, and genes associated with podocyte injury and foot process effacement.

Means of injury	Category	Agent/Molecule/Gene	References
Toxicity	Actin reorganization	Adriamycin	40– 49
		Diphtheria toxin	50– 52
		Indoxyl sulfate	53
		Puromycin aminonucleoside	6, 24– 39
		Shigatoxin	54– 56
Immunologic	Actin reorganization	Complement proteins	66– 70
		Lipopolysaccharides	57– 63
		Polyinosinic-polycytidylic acid	65
	Anti-GBM antibodies	Rabbit anti-mouse GBM antiserum	71– 74
		Rabbit anti-rat GBM antiserum	75, 76
		Sheep anti-rabbit GBM antiserum	77, 78
Charge distortion	Podocyte polarity	Poly-L-lysine	85
		Protamine sulfate	30, 79– 87
		Sialidase	11, 88
Signaling pathway activation	Actin reorganization and motility	Angiopoietin-like3 (ANGPTL3)	271
		B7-1 (CD80)	57, 272
		Cytosolic cathepsin L (cCATL)	61– 64
		Glucose	273– 277
		Glutamine	64
		Insulin	227
		Integrin β3 (ITGB3)	60, 278
		Tumor necrosis factor-α (TNF-α)	279– 281
		Transient receptor potential cation channel 5/6 (TRPC5 and TRPC6)	282
		Urokinase-type plasminogen activator receptor (uPAR)	60
	Apoptosis and mitochondrial dysfunction	Albumin	283– 285
		Aldosterone	286– 288
		Angiopoietin-like 3 (ANGPTL3)	289
		Angiotensin II	290– 294
		Fatty acids	295– 297
		Glucose	232, 294, 298
		IGF-binding protein-3 (IGFBP-3)	299
		Oxidized low-density lipoprotein (LDL)	300
		Transforming growth factor-β1 (TGF-β1)	237, 301– 304
	Autophagy	Angiotensin II	305
		Glucose	306
	EMT	Endothelin-1 (ET-1)	263
		Integrin-linked kinase (ILK)	212
		Puromycin aminonucleoside	262
		Transforming growth factor-β1 (TGF-β1)	308
	Proliferative	Negative factor (NEF)	309, 310
		Tumor necrosis factor-α (TNF-α)	311
	Proteinuric	Hypo-sialylated angiopoietin-like 4 (ANGPTL4)	32
		Soluble urokinase-type plasminogen activator receptor (suPAR)	278
	Anti-proteinuric	Circulating sialylated angiopoietin-like 4 (ANGPTL4)	312
	Survival	Activated protein C (APC)	313, 314
		Bone morphogenetic protein-7 (BMP7)	277, 299, 315
		Insulin-like growth factor-II (IGF-II)	228
		Vascular endothelial growth factor A (VEGF-A)	316– 318
		Vascular endothelial growth factor C (VEGF-C)	318, 319
Genetic modification	Actin-regulating proteins and enzymes	aarF domain containing kinase 4 ( *ADCK4*)	99
		α-actinin 4 ( *ACTN4*)	93– 96
		Anillin ( *ANLN*)	100
		Apolipoprotein L1 ( *APOL1*)	101
		RhoA-activated Rac1 GTPase-activating protein 24 ( *ARHGAP24*)	102
		Rho guanine nucleotide dissociation inhibitor-α ( *ARHGDIA*)	103– 105
		Claudin-1 ( *CLDN1*)	106
		Chloride intracellular channel 5A ( *CLIC5A*)	107
		Cofilin-1 ( *CFL1*)	108, 109
		Dynamin ( *DYN*)	61, 97, 98
		Ezrin ( *EZR*)	110
		Inverted formin 2 ( *INF2*)	111– 114
		Kidney ankyrin repeat-containing protein ( *KANK1*, *KANK2*, and *KANK4*)	115
		Neuronal Wiskott-Aldrich syndrome protein ( *N-WASP*)	116
		Class II phosphoinositide 3-kinase C2 α ( *PI3KC2α*)	117
		Phospholipase C ε1 ( *PLCE1*)	118
		Rac1 ( *RAC1*)	119, 120
		Rhophilin 1 ( *RHPN1*)	121
		Schwannomin interacting protein 1 ( *SCHIP1*)	122
		Synaptopodin ( *SYNPO*)	90– 92
		WT1-interacting protein ( *WTIP*)	123
	Lysosomal protein	Lysosome membrane protein 2 ( *SCARB2/LIMP2*)	190
	Mitochondrial proteins	Coenzyme Q _2_ ( *COQ2*)	191
		Coenzyme Q _6_ ( *COQ6*)	192
		Mpv17 ( *MPV17*)	193
		Mitochondrial tRNA leucine 1 ( *MTTL1*)	194
		Prohibitin ring complex subunit prohibitin-2 ( *PHB2*)	195
	Transcription factors	C-Maf-inducing protein ( *CMIP*)	242, 243
		Forkhead box C2 ( *FOXC2*)	244
		Hypoxia-inducible factor 1 α ( *HIF1A*)	245– 247
		Krüppel-like factor 6 ( *KLF6*)	248
		LIM homeobox transcription factor 1 β ( *LMX1B*)	249– 254
		V-Maf avian musculoaponeurotic fibrosarcoma oncogene homolog B ( *MAFB*)	255, 256
		Nuclear factor of activated T cells ( *NFAT*)	257, 258
		Paired box gene 2 ( *PAX2*)	259
		Podocyte-expressed 1/Transcription factor 21 ( *POD1/TCF21*)	260
		Peroxisome proliferator-activated receptor-α (PPARA)	261
		Snail family zinc finger 1 ( *SNAI1*)	262, 263
		Wilm’s tumor 1 ( *WT-1*)	264– 268
		Zinc finger E-box-binding homeobox 2 ( *ZEB2*)	269
		Zinc fingers and homeoboxes 1/2/3 ( *ZHX1, ZHX2, ZHX3*)	270
	Apoptosis and survival	Dendrin ( *DDN*)	203
		Survivin ( *BIRC5*)	204
		Vascular endothelial growth factor A ( *VEGF-A*)	207, 208
		Yes-associated protein ( *YAP*)	205, 206
	Autophagy regulating proteins	Autophagy-related 5 ( *ATG5*)	196
		Mammalian target of rapamycin ( *MTOR*)	197
		Prorenin receptor ( *PRR*)	198– 200
		Class III phosphoinositide 3-kinase/Vacuolar protein sorting 34 ( *PIK3C3/VPS34*)	201, 202
	Signaling pathway activation	β-catenin ( *CTNNB1*)	209
		Notch intracellular domain 1 ( *ICN1*)	210
		Integrin-linked kinase ( *ILK*)	211– 213
		Negative factor ( *NEF*)	214, 215
		Notch’s intracellular domain ( *NOTCH-IC*)	216
		Septin ( *SEPT7*)	217
		Transforming growth factor β ( *TGF-β*)	218
		Tuberous sclerosis complex 1 ( *TSC1*)	219
		Vpr-binding protein ( *VPR*)	215
		Wingless-type MMTV integration site family 1 ( *WNT1*)	220
	Signaling pathway reduction	Akt2 ( *AKT2*)	221
		PINCH-1–binding ankyrin repeat domain of ILK ( *ANK*)	222
		Angiotensin II receptor 2 ( *AT2*)	223
		β-catenin ( *CTNNB1*)	209, 224, 225
		Diaphanous interacting protein ( *DIP*)	226
		Dickkopf WNT signaling pathway inhibitor 1 ( *DKK1*)	209, 220, 225
		Insulin-like growth factor-I receptor ( *IGF-IR*)	195, 227– 229
		Insulin receptor ( *INSR*)	195, 227, 229
		NF-κB essential modulator ( *NEMO*)	230, 231
		Notch-1 ( *NOTCH1*)	232
		Notch-3 ( *NOTCH3*)	233
		3-phosphoinositide-dependent kinase-1 ( *PDK1*)	234
		Rapamycin-sensitive adaptor protein of mTOR ( *RAPTOR*)	219, 235
		Recombining binding protein suppressor of hairless ( *RBPSUH*)	216
		Rapamycin-insensitive subunit of mTOR ( *RICTOR*)	221, 235
		SH2-domain-containing inositol polyphosphate 5-phosphatase 2 ( *SHIP2*)	236
		SMAD family member 2/3 ( *SMAD2, SMAD3*)	237
		Signal transducer and activator of transcription 3 ( *STAT3*)	238– 240
	Slit diaphragm- associated proteins	CD2-associated protein ( *CD2AP*)	63, 92, 130, 131
		Cysteine-rich motor neuron 1 ( *CRIM1*)	142
		FAT atypical cadherin 1 ( *FAT1*)	143
		Fyn proto-oncogene ( *FYN*)	92, 144, 145
		IQ domain GTPase-activating protein 1 ( *IQGAP1*)	146, 147
		MAGUK inverted 2 ( *MAGI-2*)	148
		Myosin 1c ( *MYO1C*)	149
		Myosin 1e ( *MYO1E*)	150– 153
		Nck adaptor protein 1/2 ( *NCK1*, *NCK2*)	86, 132, 133
		Nephrin ( *NPHS1*)	124– 126
		Kin of IRRE-like 1 ( *NEPH1*)	126, 154
		Podocin ( *NPHS2*)	127– 129
		Transient receptor potential cation channel 6 ( *TRPC6*)	134– 141
		Zonula occludens 1 ( *ZO-1*)	155
	Podocyte polarity	A typical protein kinase Clambda/iota ( *aPKCλ/ι*)	161– 163
		Cdc42 ( *CDC42*)	119, 160
		Glucosamine uridine diphospho– *N*-acetylglucosamine 2-epimerase/ *N*-acetylmannosamine kinase ( *GNE*)	89
		Podocalyxin ( *PC*)	156, 157
		Protein‐tyrosine phosphatase receptor o/Glomerular epithelial protein 1 ( *PTPRO/GLEPP1*)	158, 159
		Van Gogh-like (planar cell polarity) protein 2 ( *VANGL2*)	164– 166
	GBM-associated proteins and enzymes	CD9 ( *CD9*)	171
		CD151 ( *CD151*)	172– 175
		Type IV collagen α3/α4/α5 ( *COL4A3*, *COL4A4*, and *COL4A5*)	176– 178
		Discoidin domain receptor 1 ( *DDR1*)	179
		Glypican 5 ( *GPC5*)	180
		Integrin-linked kinase ( *ILK*)	168, 181, 182
		Integrin α3 ( *ITGA3*)	27, 167
		Integrin β1 ( *ITGB1*)	168, 169
		Integrin β4 ( *ITGB4*)	170
		Laminin β2 ( *LAMB2*)	183– 186
		N-deacetylase/N-sulfotransferase ( *NDST1*)	187
		RAP1 GTPase-activating protein ( *RAP1GAP*)	188
		Talin 1 ( *TLN1*)	189

GBM, glomerular basement membrane.

A commonly used experimental model to induce glomerular proteinuria is puromycin aminonucleoside (PAN) injection into rats. Upon PAN treatment, podocytes undergo significant alterations ranging from FP effacement and cytoskeletal rearrangement to diminished levels of actin cytoskeleton- and SD-associated proteins ^24– 28^. Abnormal distribution of SD proteins ^29^ and increasing levels of tight junction proteins ^6, 30, 31^ are also reported. Rats develop proteinuria after 4 or 5 days. The early phase of proteinuria is related to the secretion of hyposialylated angiopoietin-like 4 (ANGPTL4) from podocytes, which binds avidly to the GBM and is sensitive to glucocorticoids ^32^. Later stages may be mediated by direct oxidative mechanisms. Many pharmacological agents, including dexamethasone ^33, 34^, fluvastatin ^35^, erythropoietin analog darbepoetin ^36^, mizoribine ^37^, sialic acid ^38^, and nuclear factor kappa B (NF-κB) inhibitor dehydroxymethylepoxyquinomicin (DHMEQ) ^39^, have been shown to possess the ability to reverse the reorganized stress fiber and cortical actin fiber phenotype observed after PAN treatment.

Similar structural and functional abnormalities are observed in a rat model of adriamycin (ADR) ^40– 44^. However, podocyte cytoskeleton returns to almost normal appearance by day 20 in PAN-treated rats, whereas pathological and functional changes progress and proteinuria sustains during a similar period of time in ADR-induced nephropathy. Of note, most mouse strains are not susceptible to either of these reagents except BALB/c and BALB/cJ mice, which develop severe proteinuria and progressive renal failure following ADR administration ^45, 46^. Recently, a nuclear DNA repair protein Prkdc (protein kinase, DNA-activated, catalytic peptide) was discovered to participate in the maintenance of the mitochondrial genome and prevent ADR-induced nephropathy ^47^. Simvastatin ^48^ and thiazolidinedione ^49^ also confer renoprotective phenotype in response to ADR.

Other toxins, which act on podocytes and have been used experimentally, include diphtheria toxin (DT) ^50– 52^ secreted by *Corynebacterium diphtheria*, which causes acute loss of podocytes in inducible diphtheria toxin receptor (iDTR) mice; uremic toxin indoxyl sulfate ^53^, which decreases the expression of podocyte differentiation and functional marker proteins; and hemolytic uremic Shiga toxin ^54– 56^, which mediates the release of inflammatory cytokines and vasoactive mediators while potently inhibiting protein synthesis.

These models represent irreversible glomerular damage with major (20% and above *in vivo*) podocyte depletion ^50^, which leads to progressive renal failure. If podocyte loss is less than this threshold, then podocytes have the capacity to recover the normal structure of a healthy glomerulus. Administration of lipopolysaccharides (LPS) is an example of such a reversible model ^57– 59^. LPS trigger podocyte FP effacement and transient proteinuria within 24 hours, which return to baseline after 3 days ^58^. Podocytes sense LPS by Toll-like receptor 4 (TLR-4) and this pro-inflammatory response upregulates expression of the co-stimulatory molecule B7-1 ^57^ and the urokinase-type plasminogen activator receptor (uPAR) ^60^. LPS also induces the cytosolic variant of cathepsin L (CatL) enzyme ^61^, indicating that CatL upregulation in podocytes is associated with the development of proteinuria in mice through a mechanism that involves the cleavage of large GTPase dynamin ^61^, synaptopodin ^62^, and CD2-associated protein (CD2AP) ^63^. In a recent study, we reported that the modification of intracellular pH by glutamine uptake was a protective mechanism of cultured mouse podocytes against cytosolic CatL activity, which was markedly elevated under the disease state ^64^. Treatment of cultured human podocytes with TLR-3 immunostimulant polyinosinic-polycytidylic acid (polyIC) induces CatL mRNA and simultaneously downregulates podocyte marker protein synaptopodin ^65^, suggesting that polyIC may follow an injury pathway similar to that of LPS.

Another means of podocyte injury are subepithelial immune complexes developing as a result of circulating antibodies, which damage or activate podocytes through complement-dependent processes. A number of signaling pathways have been implicated in complement-mediated podocyte injury ^66– 69^, in which sublethal concentrations of complement produce a pronounced but reversible disruption of the actin cytoskeleton and associated focal contacts ^70^. Other intracellular events include endoplasmic reticulum (ER) stress, production of ROS, and proteases. Focusing on immunologically induced glomerular injury, podocytes also respond to immunologic processes particularly targeting GBM. Passive administration of heterologous sera containing cross-reacting antibodies against the GBM results in vacuolization of podocytes, focal detachment of podocytes from GBM, and immediate onset of glomerulosclerosis with crescent formation ^71– 78^ consistent with a crosstalk between podocytes and the immune system.

Distortion of glomerular charge selectivity by neutralization of the negative charges on podocytes and SDs with polycation protamine sulfate (PS) causes FPs to broaden *in vivo* ^30, 79– 81^ and stress fibers to disintegrate *in vitro* ^82^ in a calcium-dependent manner ^83, 84^. These physiological changes happen within 15 minutes following PS treatment and can be reversed by reperfusion with heparin for another 15 minutes ^81, 85, 86^. PS is also responsible for the phosphorylation of SD protein nephrin ^81, 86^ and focal adhesion complex protein Cas ^81^. On the other hand, protamine had little or no effect on the sieving coefficient (also referred to as fractional clearance) of bovine serum albumin once added to neutralize GBM polyanions, a finding that downplays the contribution of GBM to the charge selectivity exhibited by the glomerular filtration barrier ^87^. A similar structural alteration can be induced by polycation poly-L-lysine ^85^, by removal of the sialic acid ^11, 88^, or by mutation in glucosamine (UDP- *N*-acetyl)-2-epimerase/ *N*-acetylmannosamine kinase ( *GNE*), the rate-limiting enzyme of sialic acid biosynthesis ^89^. Patients with mutations in *GNE*, however, develop the rare muscle disease HIBM (hereditary inclusion body myopathy) and never get kidney disease ^89^, highlighting the differences between mice and humans in this pathway in the kidney.

Mutations, abnormalities, or genetic overexpression or deletion in genes encoding podocyte proteins, which are the regulators of actin cytoskeleton such as synaptopodin ( *SYNPO*) ^90– 92^, α-actinin 4 ( *ACTN4*) ^93– 96^, dynamin ( *DYN*) ^61, 97, 98^, aarF domain-containing kinase 4 ( *ADCK4*) ^99^, anillin ( *ANLN*) ^100^, apolipoprotein L1 ( *APOL1*) ^101^, RhoA-activated Rac1 GTPase-activating protein 24 ( *ARHGAP24*) ^102^, Rho guanine nucleotide dissociation inhibitor-α ( *ARHGDIA*) ^103– 105^, claudin-1 ( *CLDN1*) ^106^, chloride intracellular channel 5A ( *CLIC5A*) ^107^, cofilin-1 ( *CFL1*) ^108, 109^, ezrin ( *EZR*) ^110^, inverted formin 2 ( *INF2*) ^111– 114^, kidney ankyrin repeat-containing protein ( *KANK1*, *KANK2*, *KANK4*) ^115^, neuronal Wiskott-Aldrich syndrome protein (N-WASP) ^116^, class II phosphoinositide 3-kinase C2 α ( *PI3KC2α*) ^117^, phospholipase C ε1 ( *PLCE1*) ^118^, Rho family small GTP-binding protein Rac1 ( *RAC1*) ^119, 120^, rhophilin 1 ( *RHPN1*) ^121^, schwannomin interacting protein 1 ( *SCHIP1*) ^122^, and WT1-interacting protein ( *WTIP*) ^123^, and the ones that are associated with SD complex, including nephrin ( *NPHS1*) ^124– 126^, podocin ( *NPHS2*) ^127– 129^, CD2-associated protein ( *CD2AP*) ^63, 92, 130, 131^, Nck adaptor protein 1/2 ( *NCK1*, *NCK2*) ^86, 132, 133^, transient receptor potential cation channel 6 ( *TRPC6*) ^134– 141^, cysteine-rich motor neuron 1 ( *CRIM1*) ^142^, FAT atypical cadherin 1 ( *FAT1*) ^143^, Fyn proto-oncogene ( *FYN*) ^92, 144, 145^, IQ domain GTPase-activating protein 1 ( *IQGAP1*) ^146, 147^, MAGUK Inverted 2 ( *MAGI-2*) ^148^, myosin 1c ( *MYO1C*) ^149^, myosin 1e ( *MYO1E*) ^150– 153^, kin of IRRE like 1 ( *NEPH1*) ^126, 154^, and zonula occludens 1 ( *ZO-1*) ^155^, leads to proteinuric diseases owing to the disruption of filtration barrier and rearrangement of actin cytoskeleton.

Likewise, glomerular filtration barrier is impaired if the podocyte apical membrane domain proteins maintaining the negative surface charge are lost or transferred including podocalyxin ( *PC*) ^156, 157^, protein-tyrosine phosphatase receptor o/glomerular epithelial protein 1 ( *PTPRO/GLEPP1*) ^158, 159^, cdc42 ( *CDC42*) ^119, 160^, atypical protein kinase Clambda/iota ( *aPKCλ/ι*) ^161– 163^, glucosamine uridine diphospho– *N*-acetylglucosamine-2-epimerase/ *N*-acetylmannosamine kinase ( *GNE*) ^89^, and Van Gogh-like (planar cell polarity) protein 2 ( *VANGL2*) ^164– 166^. Highlighting the importance of glomerular capillary wall assembly, manipulating or deleting the genes implicated in the adhesion of podocytes to GBM components such as integrin α3 ( *ITGA3*) ^27, 167^, integrin β1 ( *ITGB1*) ^168, 169^, integrin β4 ( *ITGB4*) ^170^, CD9 ( *CD9*) ^171^, CD151 ( *CD151*) ^172– 175^, type IV collagen α3/α4/α5 ( *COL4A3*, *COL4A4*, *COL4A5*) ^176– 178^, discoidin domain receptor 1 ( *DDR1*) ^179^, glypican 5 ( *GPC5*) ^180^, integrin-linked kinase ( *ILK*) ^168, 181, 182^, laminin β2 ( *LAMB2*) ^183– 186^, N-deacetylase/N-sulfotransferase ( *NDST1*) ^187^, RAP1 GTPase-activating protein ( *RAP1GAP*) ^188^, and talin 1 ( *TLN1*) ^189^ causes disorganization of podocyte cytoskeletal architecture, leading to deformation in glomerular filtration.

The involvement of lysosome membrane protein 2 ( *SCARB2/LIMP2*) ^190^ in the maintenance of podocyte structure and mitochondrial proteins coenzyme Q _2_ ( *COQ2*) ^191^, coenzyme Q _6_ ( *COQ6*) ^192^, Mpv17 ( *MPV17*) ^193^, mitochondrial tRNA leucine 1 ( *MTTL1*) ^194^, and prohibitin ring complex subunit prohibitin-2 ( *PHB2*) ^195^ in the redox state of podocyte is reported in genetic studies. Podocyte-specific deletion of autophagy-related 5 ( *ATG5*) ^196^, mammalian target of rapamycin ( *MTOR*) ^197^, prorenin receptor ( *PRR*) ^198– 200^, and class III phosphoinositide 3-kinase/vacuolar protein sorting 34 ( *PIK3C3/VPS34*) ^201, 202^ disrupts intracellular vesicle trafficking and impairs autophagic flux. Ablation of dendrin ( *DDN*) ^203^ improves renal survival in progressive glomerulosclerosis, whereas knockdown of survivin ( *BIRC5*) ^204^, a member of the inhibitor of apoptosis protein family, and Yes-associated protein ( *YAP*) ^205, 206^, a downstream target of Hippo kinases, induces podocyte apoptosis. Podocyte-specific knockout mice for vascular endothelial growth factor A ( *VEGF-A*) demonstrated a key role for VEGF-A signaling for the establishment and maintenance of a normal glomerular filtration barrier ^207^ as well as mesangial cell survival and differentiation ^208^.

A tremendous number of genetic studies were carried out to reveal the biological role of various pathways in podocytes. Regulatory genes, including β-catenin ( *CTNNB1*) ^209^, Notch intracellular domain 1 ( *ICN1*) ^210^, *ILK* ^211– 213^, negative factor ( *NEF*) ^214, 215^, Notch’s intracellular domain ( *NOTCH-IC*) ^216^, septin ( *SEPT7*) ^217^, transforming growth factor-β ( *TGF-β*) ^218^, tuberous sclerosis complex 1 ( *TSC1*) ^219^, vpr-binding protein ( *VPR*) ^215^, and wingless-type MMTV integration site family 1 ( *WNT1*) ^220^, are used as genetic switches to turn on (activate) a specific signaling pathway. There are also studies aiming to shut down (repress) a particular pathway targeting the genes such as Akt2 ( *AKT2*) ^221^, PINCH-1–binding ankyrin repeat domain of ILK ( *ANK*) ^222^, angiotensin II receptor 2 ( *AT2*) ^223^, *CTNNB1* ^209, 224, 225^, diaphanous interacting protein ( *DIP*) ^226^, dickkopf WNT signaling pathway inhibitor 1 ( *DKK1*) ^209, 220, 225^, insulin-like growth factor-I receptor ( *IGF-IR*) ^195, 227– 229^, insulin receptor ( *INSR*) ^195, 227, 229^, NF-κB essential modulator ( *NEMO*) ^230, 231^, Notch-1 ( *NOTCH1*) ^232^, Notch-3 ( *NOTCH3*) ^233^, 3-phosphoinositide-dependent kinase-1 ( *PDK1*) ^234^, rapamycin-sensitive adaptor protein of mTOR ( *RAPTOR*) ^219, 235^ and rapamycin-insensitive subunit of mTOR ( *RICTOR*) ^221, 235^, recombining binding protein suppressor of hairless ( *RBPSUH*) ^216^, SH2-domain-containing inositol polyphosphate 5-phosphatase 2 ( *SHIP2*) ^236^, SMAD family member 2/3 ( *SMAD2, SMAD3*) ^237^, and signal transducer and activator of transcription 3 ( *STAT3*) ^238– 240^.

Currently, there is great interest in research into transcriptional regulation of gene expression patterns during development and differentiation of podocytes ^241^. Genetic studies and analysis of mutations in genes encoding transcription factors provide a comprehensive approach in characterizing the functional role of transcription factors. Alterations in c-Maf-inducing protein ( *CMIP*) ^242, 243^, forkhead box C2 ( *FOXC2*) ^244^, hypoxia-inducible factor 1 α ( *HIF1A*) ^245– 247^, Krüppel-like factor 6 ( *KLF6*) ^248^, LIM homeobox transcription factor 1 β ( *LMX1B*) ^249– 254^, v-Maf avian musculoaponeurotic fibrosarcoma oncogene homolog B ( *MAFB*) ^255, 256^, nuclear factor of activated T cells (NFAT) ^257, 258^, paired box gene 2 ( *PAX2*) ^259^, podocyte-expressed 1/transcription factor 21 ( *POD1/TCF21*) ^260^, peroxisome proliferator-activated receptor-α ( *PPARA*) ^261^, Snail family zinc finger 1 ( *SNAI1*) ^262, 263^, Wilm’s tumor 1 ( *WT-1*) ^264– 268^, zinc finger E-box-binding homeobox 2 ( *ZEB2*) ^269^, and zinc fingers and homeoboxes 1/2/3 ( *ZHX1*, *ZHX2*, and *ZHX3*) ^270^ were studied to enforce a particular cell fate by stimulating or suppressing the related genes.

In addition to these molecules, factors, and genes implicating various means of podocyte injury, there are proteins or agents such as angiopoietin-like 3 (ANGPTL3) ^271^, B7-1 (CD80) ^57, 272^, cytosolic CatL ^61– 64^, glucose ^273– 277^, glutamine ^64^, insulin ^227^, integrin β3 (ITGB3) ^60, 278^, tumor necrosis factor-α (TNF-α) ^279– 281^, transient receptor potential cation channel 5/6 (TRPC5 and TRPC6) ^282^, and uPAR ^60^ that are involved in the regulation of podocyte cytoskeleton. In some cases, this cytoskeletal disaggregation and the associated activation of certain pathways (including tumor suppressor protein p53 and caspases) lead to podocyte loss ( *in vitro*) and detachment from GBM ( *in vivo*). Seminal studies have shown that apoptotic stimuli are mediated by albumin ^283– 285^, aldosterone ^286– 288^, angiopoietin-like3 (ANGPTL3) ^289^, angiotensin II ^290– 294^, fatty acids ^295– 297^, glucose ^232, 294, 298^, IGF-binding protein-3 (IGFBP-3) ^299^, oxidized low-density lipoprotein (LDL) ^300^, and TGF-β1 ^237, 301– 304^. Angiotensin II ^305^ and glucose ^306^ might also induce podocyte autophagic processes as evidenced by the presence of the increased number of autophagosomes and autophagic genes such as LC3-2 and beclin-1. Under specific conditions, podocyte injury leads to a phenotypic conversion, where podocytes lose their epithelial features such as nephrin, P-cadherin, and ZO-1 while acquiring mesenchymal markers such as desmin, fibroblast-specific protein-1 (FSP-1), α-smooth muscle actin (α-SMA), vimentin, type I collagen, and fibronectin ^307^. This process is referred to as podocyte’s epithelial-mesenchymal transition (EMT) and is driven in some cases by endothelin-1 (ET-1) ^263^, ILK ^212^, PAN ^262^, and TGF-β1 ^308^. When infected by human immunodeficiency virus 1 (HIV-1) NEF protein ^309, 310^ or treated by TNF-α ^311^, the podocyte gives a proliferative response marked by the loss of differentiation markers such as synaptopodin, WT-1, and GLEPP-1 and the subsequent expression of the proliferation markers such as podocyte G _1_ cyclin, cyclin A, cyclin D1, and Ki-67. There is evidence that non-cytokine-soluble factors such as soluble urokinase-type plasminogen activator receptor (suPAR) ^278^ cause podocyte FP effacement and proteinuria via a β3 integrin-dependent mechanism but that circulating sialylated ANGPTL4 ^312^ reduces proteinuria via an endothelial β5 integrin-dependent mechanism. By contrast, podocyte-secreted hypo-sialylated ANGPTL4 causes proteinuria via interactions with the GBM ^32^.

Although these complex regulatory mechanisms imply the vulnerability of podocytes, a variety of factors support podocyte differentiation and survival, including activated protein C (APC) ^313, 314^, bone morphogenetic protein-7 (BMP7) ^277, 299, 315^, insulin-like growth factor-II (IGF-II) ^228^, VEGF-A ^316– 318^, and vascular endothelial growth factor C (VEGF-C) ^318, 319^.

## Podocytes in glomerular disease pathology

Although the podocyte injury is not the only cause of major glomerular diseases, a stable podocyte architecture with interdigitating FPs connected by highly specialized filtration slits is essential for the maintenance and proper function of the glomerular filtration barrier. Both experimental and clinical studies have indicated a pivotal role of podocyte injury in the development and progression of glomerular diseases.

A number of different conditions and health risk factors can result in glomerular disease. Nephrotic syndrome or glomerulonephritis (i.e., malfunction of glomerular filter) may be a direct result of an infection or accumulation of toxic agents in kidneys, or (podocyte- and GBM-associated) genetic defects, or may be due to a secondary insult such as a pre-existing disease occurring in the body ^320– 322^. This represents the conventional approach to classification of glomerular diseases, which generally meets the needs of nephrologists.

The most common cause of primary glomerular disease in adults is focal segmental glomerulosclerosis (FSGS), which is defined by the scarring (sclerosis) of some but not all of the glomeruli (focal) that involves only a section of the affected glomeruli (segmental) by light microscopy of a renal biopsy specimen. In most cases, distinguishing primary (idiopathic) FSGS from the genetic form of FSGS associated with mutations in essential podocyte proteins ^323, 324^ or secondary FSGS (linked to a variety of conditions, including viral infections, drug toxicity, or previous glomerular injury) ^325^ is challenging; however, it has been proven that this heterogeneous lesion results from podocyte injury ^20, 50, 326, 327^. Once the integrity of podocyte FPs is lost, podocytes start to detach from underlying GBM at certain sites revealing bare areas of glomerular capillary surface. Later, these bare areas of GBM contact the Bowman’s capsule and form synechia, which represents the earliest committed FSGS lesion. This sequence of pathological events eventually leads to the development of more lesions and progression to glomerulosclerosis ^328^. Recurrence of FSGS in renal transplant recipients has given rise to the existence of permeability or circulating factor(s) acting on podocytes as the cause of primary FSGS ^329^. To date, a few plasma factors have been proposed ^330^ but most of these have been found to be non-specific to FSGS serum/plasma ^331^. Recently, suPAR was found to be associated with FSGS; for example, two thirds of patients with primary FSGS exhibited high levels of suPAR and those with the highest levels had a greater chance of recurrence after transplantation ^278^. In support of this, our group found that higher suPAR levels at baseline are independently associated with faster decline in eGFR and suPAR in plasma can predict risk of developing chronic kidney disease (CKD) in healthy people up to five years before its onset ^332^.

Contrary to FSGS, in which podocytes are lost in the areas of sclerosis, minimal change disease (MCD) is a reversible disorder with normal histology and does not cause podocyte depletion. Diffuse effacement of podocyte FPs (accompanied by condensation of the actin-based cytoskeleton but not associated with reduction of any key podocyte-specific protein except podocyte alpha-dystroglycan ^333^) and loss of GBM charge are among the classic features of MCD. All of these changes, and the development of selective proteinuria, are attributed to the secretion from podocytes of a form of ANGPTL4 that lacks sialic acid residues ^32, 334^.

An FSGS-related but morphologically distinct phenotype was observed when podocytes are infected with HIV ^335, 336^ or induced by infections, drugs, autoimmune diseases, or organ transplants ^337– 339^. This phenotype is described as collapsing glomerulopathy (CG) and is characterized by extensive loss of mature podocyte markers, severe FP effacement, and focal detachment together with the collapse of the capillary loops. Importantly, podocytes re-enter the cell cycle, become capable of proliferating, and lead to the formation of crescents filling the Bowman’s space, making CG structurally distinct from other forms of FSGS ^214, 309, 340^. If left untreated, HIV-1-associated nephropathy progresses to end-stage renal disease within weeks to months, whereas the combined antiretroviral therapy, which blocks HIV-1 replication, limits podocyte hyperplasia and hypertrophy and brings podocytes back to differentiation state ^341^. Studies using animal models have demonstrated that CG can be ameliorated by using cell-cycle inhibitors ^342^ or by activating transcription factors involved in podocyte differentiation ^343^; however, full recovery from CG is scarce ^344^.

The immunoglobulin A (IgA) nephropathy (IgAN), which is the most prevalent primary chronic glomerular disease worldwide ^345^, exhibits significant heterogeneity in terms of histopathologic features and clinical outcomes ^346^. Emerging data suggest that mesangial deposition of IgA1 immune complexes leads to podocyte necrosis and detachment from the GBM ^347, 348^ with the subsequent reduction in nephrin mRNA ^349^.

Obesity-related hypertension and diabetes have become epidemic health problems worldwide and major risk factors for the development of CKD ^350^. High glucose altered podocyte actin assembly *in vitro* ^351^, high blood glucose (hyperglycemia) induced podocyte apoptosis via the ROS-dependent pathway in obese rodents ^298^, and podocyte density and number decreased in patients with obesity-related glomerulopathy ^352^.

## Targeting podocytes as renal-specific therapy

Human kidney has been considered a terminally differentiated organ with minimal cellular turnover and limited capacity for repair, suggesting that kidney injuries carrying severe consequences have limited treatment options. The goal of clinical nephrologists and renal researchers should be to identify the renal protection mechanism and to develop strategies for the treatment of kidney or various renal compartments of which kidney is composed. Podocytes are probably the most likely candidate cell population to be analyzed on a molecular level since these intricate cells are the most vulnerable component of the glomerular filtration network even during early stages of injury and serve as hallmarks of a state of glomerular disease ^353^. Owing to their post-mitotic nature, podocytes have a limited capacity for cell division and do not regenerate in response to injury and loss ^354^. This leads to rapid progression of glomerular diseases unless treated. Regardless of the diverse origins of glomerular diseases, podocytes are critical determinants of outcome for all glomerular diseases, which makes podocytes a unique model for monitoring and investigating disease progression ^355^.

Therefore, there has been a pronounced shift toward podocyte proteins as therapeutic targets in the last decade ^356^. Sialic acid and its precursors show efficacy in MCD ^32^ and diabetic nephropathy ^357^. Mutant forms of human *ANGPTL4* reduce proteinuria without causing hypertriglyceridemia in FSGS and diabetic nephropathy ^312, 357^. Calcineurin inhibitor cyclosporine A (CsA) stabilizes of the actin cytoskeleton and stress fibers in podocytes by blocking the calcineurin-mediated phosphorylation and CatL-facilitated degradation of synaptopodin ^62^. As mentioned earlier, suPAR, which activates integrin αvβ3 independent of uPAR, has been suggested as an FSGS factor ^278^. A specific inhibitor of integrin αvβ3, cyclo-RGDfV, ameliorates proteinuria in mouse models of nephrotic syndrome by directly targeting the upregulated integrin αvβ3 on podocytes ^60^. CD20 antibody rituximab binds to sphingomyelin phosphodiesterase acid-like 3b (SMPDL-3b) and stabilizes the structure and function of podocytes treated with the sera of patients with recurrent FSGS ^358^. Abatacept blocks the interaction of B7-1 (CD80) with cytoskeletal protein talin and thereby stabilizes β1-integrin activity and prevents podocyte motility ^359^. However, these results have been subjected to criticism since uncertainties still remain, preventing us from being too optimistic about the general efficacy of abatacept ^360– 362^. The GTPase dynamin, which promotes endocytosis and regulates actin cytoskeleton ^61, 97^, is induced by small-molecule Bis-T-23 as a potential therapeutic approach ^363^. Bis-T-23 effectively promotes dynamin assembly into higher-order structures and increases actin polymerization in injured podocytes ^361^.

Molecular analysis of podocytes will lead to a better understanding of disease mechanisms and therefore may enable the identification of targets for early-onset diagnostics and disease treatment. In this context, cell-based high-throughput drug screening assays quantifying the phenotypic changes in podocytes (specifically changes in morphology, F-actin cytoskeleton, focal adhesions, cell volume, and so on) offer great value for the discovery of chemotherapeutic agents. Recently, a podocyte cell-based phenotypic assay was developed and applied to identify novel podocyte-protective small molecules and establish specific drug delivery strategies ^364^.

The possibilities of targeting podocytes and thereby affecting kidney disease and progression early in the course set high expectations and hopefully will provide a significant benefit to human health in the future.

## References

[1] Pavenstädt H Kriz W Kretzler M: Cell biology of the glomerular podocyte. *Physiol Rev.* 2003;83(1):253–307. 10.1152/physrev.00020.2002 12506131

[2] Andrews PM: Investigations of cytoplasmic contractile and cytoskeletal elements in the kidney glomerulus. *Kidney Int.* 1981;20(5):549–62. 10.1038/ki.1981.176 7201039

[3] Kriz W Hackenthal E Nobiling R: A role for podocytes to counteract capillary wall distension. *Kidney Int.* 1994;45(2):369–76. 10.1038/ki.1994.47 8164421

[4] Ichimura K Kurihara H Sakai T: Actin filament organization of foot processes in rat podocytes. *J Histochem Cytochem.* 2003;51(12):1589–600. 10.1177/002215540305101203 14623927

[5] Reiser J Kriz W Kretzler M: The glomerular slit diaphragm is a modified adherens junction. *J Am Soc Nephrol.* 2000;11(1):1–8. 10616834 10.1681/ASN.V1111

[6] Fukasawa H Bornheimer S Kudlicka K: Slit diaphragms contain tight junction proteins. *J Am Soc Nephrol.* 2009;20(7):1491–503. 10.1681/ASN.2008101117 19478094 PMC2709684

[7] Huber TB Benzing T: The slit diaphragm: a signaling platform to regulate podocyte function. *Curr Opin Nephrol Hypertens.* 2005;14(3):211–6. 10.1097/01.mnh.0000165885.85803.a8 15821412

[8] George B Holzman LB: Signaling from the podocyte intercellular junction to the actin cytoskeleton. *Semin Nephrol.* 2012;32(4):307–18. 10.1016/j.semnephrol.2012.06.002 22958485 PMC3438455

[9] Grahammer F Schell C Huber TB: The podocyte slit diaphragm--from a thin grey line to a complex signalling hub. *Nat Rev Nephrol.* 2013;9(10):587–98. 10.1038/nrneph.2013.169 23999399

[10] Kerjaschki D Sharkey DJ Farquhar MG: Identification and characterization of podocalyxin--the major sialoprotein of the renal glomerular epithelial cell. *J Cell Biol.* 1984;98(4):1591–6. 10.1083/jcb.98.4.1591 6371025 PMC2113206

[11] Gelberg H Healy L Whiteley H: *In vivo* enzymatic removal of alpha 2-->6-linked sialic acid from the glomerular filtration barrier results in podocyte charge alteration and glomerular injury. *Lab Invest.* 1996;74(5):907–20. 8642786

[12] Satchell SC Braet F: Glomerular endothelial cell fenestrations: an integral component of the glomerular filtration barrier. *Am J Physiol Renal Physiol.* 2009;296(5):F947–56. 10.1152/ajprenal.90601.2008 19129259 PMC2681366

[13] Kretzler M: Regulation of adhesive interaction between podocytes and glomerular basement membrane. *Microsc Res Tech.* 2002;57(4):247–53. 10.1002/jemt.10083 12012393

[14] Farquhar MG: The glomerular basement membrane: not gone, just forgotten. *J Clin Invest.* 2006;116(8):2090–3. 10.1172/JCI29488 16886057 PMC1523392

[15] Tryggvason K Wartiovaara J: How does the kidney filter plasma? *Physiology (Bethesda).* 2005;20(2):96–101. 10.1152/physiol.00045.2004 15772298

[16] Menon MC Chuang PY He CJ: The glomerular filtration barrier: components and crosstalk. *Int J Nephrol.* 2012;2012: 749010. 10.1155/2012/749010 22934182 PMC3426247

[17] Haraldsson B Nyström J Deen WM: Properties of the glomerular barrier and mechanisms of proteinuria. *Physiol Rev.* 2008;88(2):451–87. 10.1152/physrev.00055.2006 18391170

[18] Mundel P Reiser J: Proteinuria: an enzymatic disease of the podocyte? *Kidney Int.* 2010;77(7):571–80. 10.1038/ki.2009.424 19924101 PMC4109304

[19] Endlich K Kriz W Witzgall R: Update in podocyte biology. *Curr Opin Nephrol Hypertens.* 2001;10(3):331–40. 11342794 10.1097/00041552-200105000-00006

[20] Asanuma K Mundel P: The role of podocytes in glomerular pathobiology. *Clin Exp Nephrol.* 2003;7(4):255–9. 10.1007/s10157-003-0259-6 14712353

[21] Garg P Holzman LB: Podocytes: gaining a foothold. *Exp Cell Res.* 2012;318(9):955–63. 10.1016/j.yexcr.2012.02.030 22421512

[22] Barisoni L Schnaper HW Kopp JB: Advances in the biology and genetics of the podocytopathies: implications for diagnosis and therapy. *Arch Pathol Lab Med.* 2009;133(2):201–16. 19195964 10.1043/1543-2165-133.2.201 PMC3118840

[23] Bierzynska A Soderquest K Koziell A: Genes and podocytes - new insights into mechanisms of podocytopathy. *Front Endocrinol (Lausanne).* 2015;5:226. 10.3389/fendo.2014.00226 25667580 PMC4304234

[24] Bertram JF Messina A Ryan GB: *In vitro* effects of puromycin aminonucleoside on the ultrastructure of rat glomerular podocytes. *Cell Tissue Res.* 1990;260(3):555–63. 10.1007/BF00297236 2372813

[25] Whiteside CI Cameron R Munk S: Podocytic cytoskeletal disaggregation and basement-membrane detachment in puromycin aminonucleoside nephrosis. *Am J Pathol.* 1993;142(5):1641–53. 8494056 PMC1886918

[26] Kim YH Goyal M Kurnit D: Podocyte depletion and glomerulosclerosis have a direct relationship in the PAN-treated rat. *Kidney Int.* 2001;60(3):957–68. 10.1046/j.1523-1755.2001.060003957.x 11532090

[27] Reiser J Oh J Shirato I: Podocyte migration during nephrotic syndrome requires a coordinated interplay between cathepsin L and alpha3 integrin. *J Biol Chem.* 2004;279(33):34827–32. 10.1074/jbc.M401973200 15197181

[28] Marshall CB Pippin JW Krofft RD: Puromycin aminonucleoside induces oxidant-dependent DNA damage in podocytes *in vitro* and *in vivo*. *Kidney Int.* 2006;70(11):1962–73. 10.1038/sj.ki.5001965 17035936

[29] Fukuda H Hidaka T Takagi-Akiba M: Podocin is translocated to cytoplasm in puromycin aminonucleoside nephrosis rats and in poor-prognosis patients with IgA nephropathy. *Cell Tissue Res.* 2015;360(2):391–400. 10.1007/s00441-014-2100-9 25676004 PMC4544490

[30] Kurihara H Anderson JM Kerjaschki D: The altered glomerular filtration slits seen in puromycin aminonucleoside nephrosis and protamine sulfate-treated rats contain the tight junction protein ZO-1. *Am J Pathol.* 1992;141(4):805–16. 1415478 PMC1886648

[31] Zhao L Yaoita E Nameta M: Claudin-6 localized in tight junctions of rat podocytes. *Am J Physiol Regul Integr Comp Physiol.* 2008;294(6):R1856–62. 10.1152/ajpregu.00862.2007 18367650

[32] Clement LC Avila-Casado C Macé C: Podocyte-secreted angiopoietin-like-4 mediates proteinuria in glucocorticoid-sensitive nephrotic syndrome. *Nat Med.* 2011;17(1):117–22. 10.1038/nm.2261 21151138 PMC3021185

[33] Wada T Pippin JW Marshall CB: Dexamethasone prevents podocyte apoptosis induced by puromycin aminonucleoside: role of p53 and Bcl-2-related family proteins. *J Am Soc Nephrol.* 2005;16(9):2615–25. 10.1681/ASN.2005020142 15987750

[34] Wada T Pippin JW Nangaku M: Dexamethasone's prosurvival benefits in podocytes require extracellular signal-regulated kinase phosphorylation. *Nephron Exp Nephrol.* 2008;109(1):e8–19. 10.1159/000131892 18480613

[35] Shibata S Nagase M Fujita T: Fluvastatin ameliorates podocyte injury in proteinuric rats *via* modulation of excessive Rho signaling. *J Am Soc Nephrol.* 2006;17(3):754–64. 10.1681/ASN.2005050571 16452496

[36] Eto N Wada T Inagi R: Podocyte protection by darbepoetin: preservation of the cytoskeleton and nephrin expression. *Kidney Int.* 2007;72(4):455–63. 10.1038/sj.ki.5002311 17457371

[37] Takeuchi S Hiromura K Tomioka M: The immunosuppressive drug mizoribine directly prevents podocyte injury in puromycin aminonucleoside nephrosis. *Nephron Exp Nephrol.* 2010;116(1):e3–10. 10.1159/000314668 20502051

[38] Pawluczyk IZ Najafabadi MG Brown JR: Sialic acid supplementation ameliorates puromycin aminonucleoside nephrosis in rats. *Lab Invest.* 2015;95(9):1019–28. 10.1038/labinvest.2015.78 26121320

[39] Shimo T Adachi Y Yamanouchi S: A novel nuclear factor κB inhibitor, dehydroxymethylepoxyquinomicin, ameliorates puromycin aminonucleoside-induced nephrosis in mice. *Am J Nephrol.* 2013;37(4):302–9. 10.1159/000348803 23548793

[40] Bertani T Poggi A Pozzoni R: Adriamycin-induced nephrotic syndrome in rats: sequence of pathologic events. *Lab Invest.* 1982;46(1):16–23. 6172662

[41] Okuda S Oh Y Tsuruda H: Adriamycin-induced nephropathy as a model of chronic progressive glomerular disease. *Kidney Int.* 1986;29(2):502–10. 10.1038/ki.1986.28 3486312

[42] Rossmann P Matousovic K Bohdanecká M: Experimental adriamycin nephropathy. Fine structure, morphometry, glomerular polyanion, and cell membrane antigens. *J Pathol.* 1993;169(1):99–108. 10.1002/path.1711690115 8433220

[43] Wang Y Wang YP Tay YC: Progressive adriamycin nephropathy in mice: sequence of histologic and immunohistochemical events. *Kidney Int.* 2000;58(4):1797–804. 10.1046/j.1523-1755.2000.00342.x 11012915

[44] Zoja C Garcia PB Rota C: Mesenchymal stem cell therapy promotes renal repair by limiting glomerular podocyte and progenitor cell dysfunction in adriamycin-induced nephropathy. *Am J Physiol Renal Physiol.* 2012;303(9):F1370–81. 10.1152/ajprenal.00057.2012 22952284

[45] Chen A Wei CH Sheu LF: Induction of proteinuria by adriamycin or bovine serum albumin in the mouse. *Nephron.* 1995;69(3):293–300. 10.1159/000188473 7753263

[46] Chen A Sheu LF Ho YS: Experimental focal segmental glomerulosclerosis in mice. *Nephron.* 1998;78(4):440–52. 10.1159/000044974 9578071

[47] Papeta N Zheng Z Schon EA: Prkdc participates in mitochondrial genome maintenance and prevents Adriamycin-induced nephropathy in mice. *J Clin Invest.* 2010;120(11):4055–64. 10.1172/JCI43721 20978358 PMC2964992

[48] Zhang W Li Q Wang L: Simvastatin ameliorates glomerulosclerosis in Adriamycin-induced-nephropathy rats. *Pediatr Nephrol.* 2008;23(12):2185–94. 10.1007/s00467-008-0933-8 18791746 PMC7811526

[49] Liu HF Guo LQ Huang YY: Thiazolidinedione attenuate proteinuria and glomerulosclerosis in Adriamycin-induced nephropathy rats via slit diaphragm protection. *Nephrology (Carlton).* 2010;15(1):75–83. 10.1111/j.1440-1797.2009.01146.x 20377774

[50] Wharram BL Goyal M Wiggins JE: Podocyte depletion causes glomerulosclerosis: diphtheria toxin-induced podocyte depletion in rats expressing human diphtheria toxin receptor transgene. *J Am Soc Nephrol.* 2005;16(10):2941–52. 10.1681/ASN.2005010055 16107576

[51] Sato Y Wharram BL Lee SK: Urine podocyte mRNAs mark progression of renal disease. *J Am Soc Nephrol.* 2009;20(5):1041–52. 10.1681/ASN.2007121328 19389856 PMC2678045

[52] Goldwich A Steinkasserer A Gessner A: Impairment of podocyte function by diphtheria toxin--a new reversible proteinuria model in mice. *Lab Invest.* 2012;92(12):1674–85. 10.1038/labinvest.2012.133 23007132

[53] Ichii O Otsuka-Kanazawa S Nakamura T: Podocyte injury caused by indoxyl sulfate, a uremic toxin and aryl-hydrocarbon receptor ligand. *PLoS One.* 2014;9(9):e108448. 10.1371/journal.pone.0108448 25244654 PMC4171541

[54] Hughes AK Stricklett PK Schmid D: Cytotoxic effect of Shiga toxin-1 on human glomerular epithelial cells. *Kidney Int.* 2000;57(6):2350–9. 10.1046/j.1523-1755.2000.00095.x 10844605

[55] Morigi M Buelli S Zanchi C: Shigatoxin-induced endothelin-1 expression in cultured podocytes autocrinally mediates actin remodeling. *Am J Pathol.* 2006;169(6):1965–75. 10.2353/ajpath.2006.051331 17148661 PMC1762486

[56] Locatelli M Buelli S Pezzotta A: Shiga toxin promotes podocyte injury in experimental hemolytic uremic syndrome *via* activation of the alternative pathway of complement. *J Am Soc Nephrol.* 2014;25(8):1786–98. 10.1681/ASN.2013050450 24578132 PMC4116050

[57] Reiser J von Gersdorff G Loos M: Induction of B7-1 in podocytes is associated with nephrotic syndrome. *J Clin Invest.* 2004;113(10):1390–7. 10.1172/JCI200420402 15146236 PMC406528

[58] Sun Y He L Takemoto M: Glomerular transcriptome changes associated with lipopolysaccharide-induced proteinuria. *Am J Nephrol.* 2009;29(6):558–70. 10.1159/000191469 19136817

[59] Srivastava T Sharma M Yew KH: LPS and PAN-induced podocyte injury in an *in vitro* model of minimal change disease: changes in TLR profile. *J Cell Commun Signal.* 2013;7(1):49–60. 10.1007/s12079-012-0184-0 23161414 PMC3590361

[60] Wei C Möller CC Altintas MM: Modification of kidney barrier function by the urokinase receptor. *Nat Med.* 2008;14(1):55–63. 10.1038/nm1696 18084301

[61] Sever S Altintas MM Nankoe SR: Proteolytic processing of dynamin by cytoplasmic cathepsin L is a mechanism for proteinuric kidney disease. *J Clin Invest.* 2007;117(8):2095–104. 10.1172/JCI32022 17671649 PMC1934589

[62] Faul C Donnelly M Merscher-Gomez S: The actin cytoskeleton of kidney podocytes is a direct target of the antiproteinuric effect of cyclosporine A. *Nat Med.* 2008;14(9):931–8. 10.1038/nm.1857 18724379 PMC4109287

[63] Yaddanapudi S Altintas MM Kistler AD: CD2AP in mouse and human podocytes controls a proteolytic program that regulates cytoskeletal structure and cellular survival. *J Clin Invest.* 2011;121(10):3965–80. 10.1172/JCI58552 21911934 PMC3195478

[64] Altintas MM Moriwaki K Wei C: Reduction of proteinuria through podocyte alkalinization. *J Biol Chem.* 2014;289(25):17454–67. 10.1074/jbc.M114.568998 24817115 PMC4067184

[65] Shimada M Ishimoto T Lee PY: Toll-like receptor 3 ligands induce CD80 expression in human podocytes via an NF-κB-dependent pathway. *Nephrol Dial Transplant.* 2012;27(1):81–9. 10.1093/ndt/gfr271 21617192

[66] Cybulsky AV Quigg RJ Salant DJ: Experimental membranous nephropathy redux. *Am J Physiol Renal Physiol.* 2005;289(4):F660–71. 10.1152/ajprenal.00437.2004 16159900 PMC1325222

[67] Berger SP Daha MR: Complement in glomerular injury. *Semin Immunopathol.* 2007;29(4):375–84. 10.1007/s00281-007-0090-3 17901956 PMC2071951

[68] Noris M Remuzzi G: Overview of complement activation and regulation. *Semin Nephrol.* 2013;33(6):479–92. 10.1016/j.semnephrol.2013.08.001 24161035 PMC3820029

[69] Takano T Elimam H Cybulsky AV: Complement-mediated cellular injury. *Semin Nephrol.* 2013;33(6):586–601. 10.1016/j.semnephrol.2013.08.009 24161043

[70] Topham PS Haydar SA Kuphal R: Complement-mediated injury reversibly disrupts glomerular epithelial cell actin microfilaments and focal adhesions. *Kidney Int.* 1999;55(5):1763–75. 10.1046/j.1523-1755.1999.00407.x 10231439

[71] Le Hir M Haas C Marino M: Prevention of crescentic glomerulonephritis induced by anti-glomerular membrane antibody in tumor necrosis factor-deficient mice. *Lab Invest.* 1998;78(12):1625–31. 9881962

[72] Le Hir M Keller C Eschmann V: Podocyte bridges between the tuft and Bowman's capsule: an early event in experimental crescentic glomerulonephritis. *J Am Soc Nephrol.* 2001;12(10):2060–71. 11562404 10.1681/ASN.V12102060

[73] Moeller MJ Soofi A Hartmann I: Podocytes populate cellular crescents in a murine model of inflammatory glomerulonephritis. *J Am Soc Nephrol.* 2004;15(1):61–7. 10.1097/01.ASN.0000102468.37809.C6 14694158

[74] Wheeler J Morley AR Appleton DR: Anti-glomerular basement membrane (GBM) glomerulonephritis in the mouse: development of disease and cell proliferation. *J Exp Pathol (Oxford).* 1990;71(3):411–22. 2372417 PMC1998690

[75] Shirato I Hosser H Kimura K: The development of focal segmental glomerulosclerosis in masugi nephritis is based on progressive podocyte damage. *Virchows Arch.* 1996;429(4–5):255–73. 10.1007/BF00198342 8972762

[76] Shirato I Sakai T Kimura K: Cytoskeletal changes in podocytes associated with foot process effacement in Masugi nephritis. *Am J Pathol.* 1996;148(4):1283–96. 8644869 PMC1861509

[77] Ophascharoensuk V Pippin JW Gordon KL: Role of intrinsic renal cells versus infiltrating cells in glomerular crescent formation. *Kidney Int.* 1998;54(2):416–25. 10.1046/j.1523-1755.1998.00003.x 9690208

[78] Kim YG Alpers CE Brugarolas J: The cyclin kinase inhibitor p21 ^CIP1/WAF1^ limits glomerular epithelial cell proliferation in experimental glomerulonephritis. *Kidney Int.* 1999;55(6):2349–61. 10.1046/j.1523-1755.1999.00504.x 10354282

[79] Seiler MW Venkatachalam MA Cotran RS: Glomerular epithelium: structural alterations induced by polycations. *Science.* 1975;189(4200):390–3. 10.1126/science.1145209 1145209

[80] Kerjaschki D: Polycation-induced dislocation of slit diaphragms and formation of cell junctions in rat kidney glomeruli: the effects of low temperature, divalent cations, colchicine, and cytochalasin B. *Lab Invest.* 1978;39(5):430–40. 104090

[81] George B Verma R Soofi AA: Crk1/2-dependent signaling is necessary for podocyte foot process spreading in mouse models of glomerular disease. *J Clin Invest.* 2012;122(2):674–92. 10.1172/JCI60070 22251701 PMC3266791

[82] Reiser J Pixley FJ Hug A: Regulation of mouse podocyte process dynamics by protein tyrosine phosphatases rapid communication. *Kidney Int.* 2000;57(5):2035–42. 10.1046/j.1523-1755.2000.00070.x 10792622

[83] Rüdiger F Greger R Nitschke R: Polycations induce calcium signaling in glomerular podocytes. *Kidney Int.* 1999;56(5):1700–9. 10.1046/j.1523-1755.1999.00729.x 10571778

[84] Schaldecker T Kim S Tarabanis C: Inhibition of the TRPC5 ion channel protects the kidney filter. *J Clin Invest.* 2013;123(12):5298–309. 10.1172/JCI71165 24231357 PMC3859394

[85] Seiler MW Rennke HG Venkatachalam MA: Pathogenesis of polycation-induced alterations ("fusion") of glomerular epithelium. *Lab Invest.* 1977;36(1):48–61. 63647

[86] Verma R Kovari I Soofi A: Nephrin ectodomain engagement results in Src kinase activation, nephrin phosphorylation, Nck recruitment, and actin polymerization. *J Clin Invest.* 2006;116(5):1346–59. 10.1172/JCI27414 16543952 PMC1401486

[87] Daniels BS: Increased albumin permeability *in vitro* following alterations of glomerular charge is mediated by the cells of the filtration barrier. *J Lab Clin Med.* 1994;124(2):224–30. 8051486

[88] Andrews PM: Glomerular epithelial alterations resulting from sialic acid surface coat removal. *Kidney Int.* 1979;15(4):376–85. 10.1038/ki.1979.49 513496

[89] Galeano B Klootwijk R Manoli I: Mutation in the key enzyme of sialic acid biosynthesis causes severe glomerular proteinuria and is rescued by *N*-acetylmannosamine. *J Clin Invest.* 2007;117(6):1585–94. 10.1172/JCI30954 17549255 PMC1878529

[90] Asanuma K Kim K Oh J: Synaptopodin regulates the actin-bundling activity of alpha-actinin in an isoform-specific manner. *J Clin Invest.* 2005;115(5):1188–98. 10.1172/JCI200523371 15841212 PMC1070637

[91] Asanuma K Yanagida-Asanuma E Faul C: Synaptopodin orchestrates actin organization and cell motility via regulation of RhoA signalling. *Nat Cell Biol.* 2006;8(5):485–91. 10.1038/ncb1400 16622418

[92] Huber TB Kwoh C Wu H: Bigenic mouse models of focal segmental glomerulosclerosis involving pairwise interaction of CD2AP, Fyn, and synaptopodin. *J Clin Invest.* 2006;116(5):1337–45. 10.1172/JCI27400 16628251 PMC1440708

[93] Kaplan JM Kim SH North KN: Mutations in ACTN4, encoding alpha-actinin-4, cause familial focal segmental glomerulosclerosis. *Nat Genet.* 2000;24(3):251–6. 10.1038/73456 10700177

[94] Kos CH Le TC Sinha S: Mice deficient in alpha-actinin-4 have severe glomerular disease. *J Clin Invest.* 2003;111(11):1683–90. 10.1172/JCI200317988 12782671 PMC156110

[95] Michaud J Lemieux LI Dubé M: Focal and segmental glomerulosclerosis in mice with podocyte-specific expression of mutant alpha-actinin-4. *J Am Soc Nephrol.* 2003;14(5):1200–11. 10.1097/01.ASN.0000059864.88610.5E 12707390

[96] Michaud JL Chaisson KM Parks RJ: FSGS-associated alpha-actinin-4 (K256E) impairs cytoskeletal dynamics in podocytes. *Kidney Int.* 2006;70(6):1054–61. 10.1038/sj.ki.5001665 16837921

[97] Gu C Yaddanapudi S Weins A: Direct dynamin-actin interactions regulate the actin cytoskeleton. *EMBO J.* 2010;29(21):3593–606. 10.1038/emboj.2010.249 20935625 PMC2982766

[98] Soda K Balkin DM Ferguson SM: Role of dynamin, synaptojanin, and endophilin in podocyte foot processes. *J Clin Invest.* 2012;122(12):4401–11. 10.1172/JCI65289 23187129 PMC3533561

[99] Ashraf S Gee HY Woerner S: *ADCK4* mutations promote steroid-resistant nephrotic syndrome through CoQ _10_ biosynthesis disruption. *J Clin Invest.* 2013;123(12):5179–89. 10.1172/JCI69000 24270420 PMC3859425

[100] Gbadegesin RA Hall G Adeyemo A: Mutations in the gene that encodes the F-actin binding protein anillin cause FSGS. *J Am Soc Nephrol.* 2014;25(9):1991–2002. 10.1681/ASN.2013090976 24676636 PMC4147982

[101] Lan X Jhaveri A Cheng K: APOL1 risk variants enhance podocyte necrosis through compromising lysosomal membrane permeability. *Am J Physiol Renal Physiol.* 2014;307(3):F326–36. 10.1152/ajprenal.00647.2013 24899058 PMC4121568

[102] Akilesh S Suleiman H Yu H: Arhgap24 inactivates Rac1 in mouse podocytes, and a mutant form is associated with familial focal segmental glomerulosclerosis. *J Clin Invest.* 2011;121(10):4127–37. 10.1172/JCI46458 21911940 PMC3195463

[103] Togawa A Miyoshi J Ishizaki H: Progressive impairment of kidneys and reproductive organs in mice lacking Rho GDIalpha. *Oncogene.* 1999;18(39):5373–80. 10.1038/sj.onc.1202921 10498891

[104] Gee HY Saisawat P Ashraf S: *ARHGDIA* mutations cause nephrotic syndrome via defective RHO GTPase signaling. *J Clin Invest.* 2013;123(8):3243–53. 10.1172/JCI69134 23867502 PMC3726174

[105] Gupta IR Baldwin C Auguste D: *ARHGDIA*: a novel gene implicated in nephrotic syndrome. *J Med Genet.* 2013;50(5):330–8. 10.1136/jmedgenet-2012-101442 23434736 PMC3625828

[106] Hasegawa K Wakino S Simic P: Renal tubular Sirt1 attenuates diabetic albuminuria by epigenetically suppressing Claudin-1 overexpression in podocytes. *Nat Med.* 2013;19(11):1496–504. 10.1038/nm.3363 24141423 PMC4041199

[107] Wegner B Al-Momany A Kulak SC: CLIC5A, a component of the ezrin-podocalyxin complex in glomeruli, is a determinant of podocyte integrity. *Am J Physiol Renal Physiol.* 2010;298(6):F1492–503. 10.1152/ajprenal.00030.2010 20335315 PMC5510016

[108] Garg P Verma R Cook L: Actin-depolymerizing factor cofilin-1 is necessary in maintaining mature podocyte architecture. *J Biol Chem.* 2010;285(29):22676–88. 10.1074/jbc.M110.122929 20472933 PMC2903407

[109] Ashworth S Teng B Kaufeld J: Cofilin-1 inactivation leads to proteinuria--studies in zebrafish, mice and humans. *PLoS One.* 2010;5(9):e12626. 10.1371/journal.pone.0012626 20838616 PMC2935884

[110] Wasik AA Koskelainen S Hyvönen ME: Ezrin is down-regulated in diabetic kidney glomeruli and regulates actin reorganization and glucose uptake via GLUT1 in cultured podocytes. *Am J Pathol.* 2014;184(6):1727–39. 10.1016/j.ajpath.2014.03.002 24726496

[111] Brown EJ Schlöndorff JS Becker DJ: Mutations in the formin gene *INF2* cause focal segmental glomerulosclerosis. *Nat Genet.* 2010;42(1):72–6. 10.1038/ng.505 20023659 PMC2980844

[112] Boyer O Benoit G Gribouval O: Mutations in *INF2* are a major cause of autosomal dominant focal segmental glomerulosclerosis. *J Am Soc Nephrol.* 2011;22(2):239–45. 10.1681/ASN.2010050518 21258034 PMC3029896

[113] Barua M Brown EJ Charoonratana VT: Mutations in the *INF2* gene account for a significant proportion of familial but not sporadic focal and segmental glomerulosclerosis. *Kidney Int.* 2013;83(2):316–22. 10.1038/ki.2012.349 23014460 PMC3647680

[114] Sun H Al-Romaih KI MacRae CA: Human Kidney Disease-causing *INF2* Mutations Perturb Rho/Dia Signaling in the Glomerulus. *EBioMedicine.* 2014;1(2–3):107–15. 10.1016/j.ebiom.2014.11.009 26086034 PMC4457406

[115] Gee HY Zhang F Ashraf S: *KANK* deficiency leads to podocyte dysfunction and nephrotic syndrome. *J Clin Invest.* 2015;125(6):2375–84. 10.1172/JCI79504 25961457 PMC4497755

[116] Schell C Baumhakl L Salou S: N-wasp is required for stabilization of podocyte foot processes. *J Am Soc Nephrol.* 2013;24(5):713–21. 10.1681/ASN.2012080844 23471198 PMC3636796

[117] Harris DP Vogel P Wims M: Requirement for class II phosphoinositide 3-kinase C2alpha in maintenance of glomerular structure and function. *Mol Cell Biol.* 2011;31(1):63–80. 10.1128/MCB.00468-10 20974805 PMC3019860

[118] Hinkes B Wiggins RC Gbadegesin R: Positional cloning uncovers mutations in *PLCE1* responsible for a nephrotic syndrome variant that may be reversible. *Nat Genet.* 2006;38(12):1397–405. 10.1038/ng1918 17086182

[119] Blattner SM Hodgin JB Nishio M: Divergent functions of the Rho GTPases Rac1 and Cdc42 in podocyte injury. *Kidney Int.* 2013;84(5):920–30. 10.1038/ki.2013.175 23677246 PMC3815690

[120] Ishizaka M Gohda T Takagi M: Podocyte-specific deletion of Rac1 leads to aggravation of renal injury in STZ-induced diabetic mice. *Biochem Biophys Res Commun.* 2015;467(3):549–55. 10.1016/j.bbrc.2015.09.158 26435502

[121] Lal MA Andersson AC Katayama K: Rhophilin-1 is a key regulator of the podocyte cytoskeleton and is essential for glomerular filtration. *J Am Soc Nephrol.* 2015;26(3):647–62. 10.1681/ASN.2013111195 25071083 PMC4341472

[122] Perisic L Rodriguez PQ Hultenby K: Schip1 is a novel podocyte foot process protein that mediates actin cytoskeleton rearrangements and forms a complex with Nherf2 and ezrin. *PLoS One.* 2015;10(3):e0122067. 10.1371/journal.pone.0122067 25807495 PMC4373682

[123] Kim JH Mukherjee A Madhavan SM: WT1-interacting protein (Wtip) regulates podocyte phenotype by cell-cell and cell-matrix contact reorganization. *Am J Physiol Renal Physiol.* 2012;302(1):F103–15. 10.1152/ajprenal.00419.2011 21900451 PMC3251346

[124] Kestilä M Lenkkeri U Männikkö M: Positionally cloned gene for a novel glomerular protein--nephrin--is mutated in congenital nephrotic syndrome. *Mol Cell.* 1998;1(4):575–82. 10.1016/S1097-2765(00)80057-X 9660941

[125] Putaala H Soininen R Kilpeläinen P: The murine nephrin gene is specifically expressed in kidney, brain and pancreas: inactivation of the gene leads to massive proteinuria and neonatal death. *Hum Mol Genet.* 2001;10(1):1–8. 10.1093/hmg/10.1.1 11136707

[126] Garg P Verma R Nihalani D: Neph1 cooperates with nephrin to transduce a signal that induces actin polymerization. *Mol Cell Biol.* 2007;27(24):8698–712. 10.1128/MCB.00948-07 17923684 PMC2169393

[127] Boute N Gribouval O Roselli S: *NPHS2*, encoding the glomerular protein podocin, is mutated in autosomal recessive steroid-resistant nephrotic syndrome. *Nat Genet.* 2000;24(4):349–54. 10.1038/74166 10742096

[128] Huber TB Simons M Hartleben B: Molecular basis of the functional podocin-nephrin complex: mutations in the *NPHS2* gene disrupt nephrin targeting to lipid raft microdomains. *Hum Mol Genet.* 2003;12(24):3397–405. 10.1093/hmg/ddg360 14570703

[129] Roselli S Heidet L Sich M: Early glomerular filtration defect and severe renal disease in podocin-deficient mice. *Mol Cell Biol.* 2004;24(2):550–60. 10.1128/MCB.24.2.550-560.2004 14701729 PMC343810

[130] Shih NY Li J Karpitskii V: Congenital nephrotic syndrome in mice lacking CD2-associated protein. *Science.* 1999;286(5438):312–5. 10.1126/science.286.5438.312 10514378

[131] Kim JM Wu H Green G: CD2-associated protein haploinsufficiency is linked to glomerular disease susceptibility. *Science.* 2003;300(5623):1298–300. 10.1126/science.1081068 12764198

[132] Jones N Blasutig IM Eremina V: Nck adaptor proteins link nephrin to the actin cytoskeleton of kidney podocytes. *Nature.* 2006;440(7085):818–23. 10.1038/nature04662 16525419

[133] Jones N New LA Fortino MA: Nck proteins maintain the adult glomerular filtration barrier. *J Am Soc Nephrol.* 2009;20(7):1533–43. 10.1681/ASN.2009010056 19443634 PMC2709686

[134] Hofstra JM Lainez S van Kuijk WH: New TRPC6 gain-of-function mutation in a non-consanguineous Dutch family with late-onset focal segmental glomerulosclerosis. *Nephrol Dial Transplant.* 2013;28(7):1830–8. 10.1093/ndt/gfs572 23291369

[135] Reiser J Polu KR Möller CC: TRPC6 is a glomerular slit diaphragm-associated channel required for normal renal function. *Nat Genet.* 2005;37(7):739–44. 10.1038/ng1592 15924139 PMC1360984

[136] Winn MP Conlon PJ Lynn KL: A mutation in the *TRPC6* cation channel causes familial focal segmental glomerulosclerosis. *Science.* 2005;308(5729):1801–4. 10.1126/science.1106215 15879175

[137] Möller CC Wei C Altintas MM: Induction of *TRPC6* channel in acquired forms of proteinuric kidney disease. *J Am Soc Nephrol.* 2007;18(1):29–36. 10.1681/ASN.2006091010 17167110

[138] Heeringa SF Möller CC Du J: A novel *TRPC6* mutation that causes childhood FSGS. *PLoS One.* 2009;4(11):e7771. 10.1371/journal.pone.0007771 19936226 PMC2777406

[139] Schlöndorff J Del Camino D Carrasquillo R: *TRPC6* mutations associated with focal segmental glomerulosclerosis cause constitutive activation of NFAT-dependent transcription. *Am J Physiol Cell Physiol.* 2009;296(3):C558–69. 10.1152/ajpcell.00077.2008 19129465 PMC2660257

[140] Chiluiza D Krishna S Schumacher VA: Gain-of-function mutations in transient receptor potential C6 (TRPC6) activate extracellular signal-regulated kinases 1/2 (ERK1/2). *J Biol Chem.* 2013;288(25):18407–20. 10.1074/jbc.M113.463059 23645677 PMC3689983

[141] Kistler AD Singh G Altintas MM: Transient receptor potential channel 6 (TRPC6) protects podocytes during complement-mediated glomerular disease. *J Biol Chem.* 2013;288(51):36598–609. 10.1074/jbc.M113.488122 24194522 PMC3868772

[142] Nyström J Hultenby K Ek S: CRIM1 is localized to the podocyte filtration slit diaphragm of the adult human kidney. *Nephrol Dial Transplant.* 2009;24(7):2038–44. 10.1093/ndt/gfn743 19158190 PMC2698089

[143] Ciani L Patel A Allen ND: Mice lacking the giant protocadherin mFAT1 exhibit renal slit junction abnormalities and a partially penetrant cyclopia and anophthalmia phenotype. *Mol Cell Biol.* 2003;23(10):3575–82. 10.1128/MCB.23.10.3575-3582.2003 12724416 PMC164754

[144] Yu CC Yen TS Lowell CA: Lupus-like kidney disease in mice deficient in the Src family tyrosine kinases Lyn and Fyn. *Curr Biol.* 2001;11(1):34–8. 10.1016/S0960-9822(00)00024-5 11166177

[145] Verma R Wharram B Kovari I: Fyn binds to and phosphorylates the kidney slit diaphragm component Nephrin. *J Biol Chem.* 2003;278(23):20716–23. 10.1074/jbc.M301689200 12668668

[146] Rigothier C Auguste P Welsh GI: IQGAP1 interacts with components of the slit diaphragm complex in podocytes and is involved in podocyte migration and permeability *in vitro*. *PLoS One.* 2012;7(5):e37695. 10.1371/journal.pone.0037695 22662192 PMC3360763

[147] Liu Y Liang W Yang Y: IQGAP1 regulates actin cytoskeleton organization in podocytes through interaction with nephrin. *Cell Signal.* 2015;27(4):867–77. 10.1016/j.cellsig.2015.01.015 25652011 PMC4356988

[148] Balbas MD Burgess MR Murali R: MAGI-2 scaffold protein is critical for kidney barrier function. *Proc Natl Acad Sci U S A.* 2014;111(41):14876–81. 10.1073/pnas.1417297111 25271328 PMC4205655

[149] Arif E Wagner MC Johnstone DB: Motor protein Myo1c is a podocyte protein that facilitates the transport of slit diaphragm protein Neph1 to the podocyte membrane. *Mol Cell Biol.* 2011;31(10):2134–50. 10.1128/MCB.05051-11 21402783 PMC3133353

[150] Krendel M Kim SV Willinger T: Disruption of Myosin 1e promotes podocyte injury. *J Am Soc Nephrol.* 2009;20(1):86–94. 10.1681/ASN.2007111172 19005011 PMC2615733

[151] Mele C Iatropoulos P Donadelli R: *MYO1E* mutations and childhood familial focal segmental glomerulosclerosis. *N Engl J Med.* 2011;365(4):295–306. 10.1056/NEJMoa1101273 21756023 PMC3701523

[152] Sanna-Cherchi S Burgess KE Nees SN: Exome sequencing identified *MYO1E* and *NEIL1* as candidate genes for human autosomal recessive steroid-resistant nephrotic syndrome. *Kidney Int.* 2011;80(4):389–96. 10.1038/ki.2011.148 21697813

[153] Chase SE Encina CV Stolzenburg LR: Podocyte-specific knockout of myosin 1e disrupts glomerular filtration. *Am J Physiol Renal Physiol.* 2012;303(7):F1099–106. 10.1152/ajprenal.00251.2012 22811491 PMC3469681

[154] Donoviel DB Freed DD Vogel H: Proteinuria and perinatal lethality in mice lacking NEPH1, a novel protein with homology to NEPHRIN. *Mol Cell Biol.* 2001;21(14):4829–36. 10.1128/MCB.21.14.4829-4836.2001 11416156 PMC87176

[155] Itoh M Nakadate K Horibata Y: The structural and functional organization of the podocyte filtration slits is regulated by Tjp1/ZO-1. *PLoS One.* 2014;9(9):e106621. 10.1371/journal.pone.0106621 25184792 PMC4153657

[156] Doyonnas R Kershaw DB Duhme C: Anuria, omphalocele, and perinatal lethality in mice lacking the CD34-related protein podocalyxin. *J Exp Med.* 2001;194(1):13–27. 10.1084/jem.194.1.13 11435469 PMC2193439

[157] Fukasawa H Obayashi H Schmieder S: Phosphorylation of podocalyxin (Ser415) Prevents RhoA and ezrin activation and disrupts its interaction with the actin cytoskeleton. *Am J Pathol.* 2011;179(5):2254–65. 10.1016/j.ajpath.2011.07.046 21945805 PMC3204015

[158] Wharram BL Goyal M Gillespie PJ: Altered podocyte structure in GLEPP1 (Ptpro)-deficient mice associated with hypertension and low glomerular filtration rate. *J Clin Invest.* 2000;106(10):1281–90. 10.1172/JCI7236 11086029 PMC381432

[159] Ozaltin F Ibsirlioglu T Taskiran EZ: Disruption of *PTPRO* causes childhood-onset nephrotic syndrome. *Am J Hum Genet.* 2011;89(1):139–47. 10.1016/j.ajhg.2011.05.026 21722858 PMC3135805

[160] Scott RP Hawley SP Ruston J: Podocyte-specific loss of Cdc42 leads to congenital nephropathy. *J Am Soc Nephrol.* 2012;23(7):1149–54. 10.1681/ASN.2011121206 22518006 PMC3380653

[161] Hirose T Satoh D Kurihara H: An essential role of the universal polarity protein, aPKClambda, on the maintenance of podocyte slit diaphragms. *PLoS One.* 2009;4(1):e4194. 10.1371/journal.pone.0004194 19142224 PMC2614475

[162] Huber TB Hartleben B Winkelmann K: Loss of podocyte aPKClambda/iota causes polarity defects and nephrotic syndrome. *J Am Soc Nephrol.* 2009;20(4):798–806. 10.1681/ASN.2008080871 19279126 PMC2663834

[163] Satoh D Hirose T Harita Y: aPKCλ maintains the integrity of the glomerular slit diaphragm through trafficking of nephrin to the cell surface. *J Biochem.* 2014;156(2):115–28. 10.1093/jb/mvu022 24700503 PMC4112437

[164] Babayeva S Zilber Y Torban E: Planar cell polarity pathway regulates actin rearrangement, cell shape, motility, and nephrin distribution in podocytes. *Am J Physiol Renal Physiol.* 2011;300(2):F549–60. 10.1152/ajprenal.00566.2009 20534871

[165] Babayeva S Rocque B Aoudjit L: Planar cell polarity pathway regulates nephrin endocytosis in developing podocytes. *J Biol Chem.* 2013;288(33):24035–48. 10.1074/jbc.M113.452904 23824190 PMC3745348

[166] Rocque BL Babayeva S Li J: Deficiency of the planar cell polarity protein Vangl2 in podocytes affects glomerular morphogenesis and increases susceptibility to injury. *J Am Soc Nephrol.* 2015;26(3):576–86. 10.1681/ASN.2014040340 25145929 PMC4341486

[167] Kreidberg JA Donovan MJ Goldstein SL: Alpha 3 beta 1 integrin has a crucial role in kidney and lung organogenesis. *Development.* 1996;122(11):3537–47. 8951069 10.1242/dev.122.11.3537

[168] Kanasaki K Kanda Y Palmsten K: Integrin beta1-mediated matrix assembly and signaling are critical for the normal development and function of the kidney glomerulus. *Dev Biol.* 2008;313(2):584–93. 10.1016/j.ydbio.2007.10.047 18082680 PMC3947521

[169] Pozzi A Jarad G Moeckel GW: Beta1 integrin expression by podocytes is required to maintain glomerular structural integrity. *Dev Biol.* 2008;316(2):288–301. 10.1016/j.ydbio.2008.01.022 18328474 PMC2396524

[170] Kambham N Tanji N Seigle RL: Congenital focal segmental glomerulosclerosis associated with beta4 integrin mutation and epidermolysis bullosa. *Am J Kidney Dis.* 2000;36(1):190–6. 10.1053/ajkd.2000.8293 10873890

[171] Blumenthal A Giebel J Warsow G: Mechanical stress enhances CD9 expression in cultured podocytes. *Am J Physiol Renal Physiol.* 2015;308(6):F602–13. 10.1152/ajprenal.00190.2014 25503725

[172] Karamatic Crew V Burton N Kagan A: CD151, the first member of the tetraspanin (TM4) superfamily detected on erythrocytes, is essential for the correct assembly of human basement membranes in kidney and skin. *Blood.* 2004;104(8):2217–23. 10.1182/blood-2004-04-1512 15265795

[173] Sachs N Kreft M van den Bergh Weerman MA: Kidney failure in mice lacking the tetraspanin CD151. *J Cell Biol.* 2006;175(1):33–9. 10.1083/jcb.200603073 17015618 PMC2064491

[174] Baleato RM Guthrie PL Gubler MC: Deletion of *CD151* results in a strain-dependent glomerular disease due to severe alterations of the glomerular basement membrane. *Am J Pathol.* 2008;173(4):927–37. 10.2353/ajpath.2008.071149 18787104 PMC2543062

[175] Blumenthal A Giebel J Ummanni R: Morphology and migration of podocytes are affected by CD151 levels. *Am J Physiol Renal Physiol.* 2012;302(10):F1265–77. 10.1152/ajprenal.00468.2011 22338088

[176] Hudson BG Tryggvason K Sundaramoorthy M: Alport's syndrome, Goodpasture's syndrome, and type IV collagen. *N Engl J Med.* 2003;348(25):2543–56. 10.1056/NEJMra022296 12815141

[177] Hudson BG: The molecular basis of Goodpasture and Alport syndromes: beacons for the discovery of the collagen IV family. *J Am Soc Nephrol.* 2004;15(10):2514–27. 10.1097/01.ASN.0000141462.00630.76 15466256

[178] Gubler MC: Inherited diseases of the glomerular basement membrane. *Nat Clin Pract Nephrol.* 2008;4(1):24–37. 10.1038/ncpneph0671 18094725

[179] Gross O Beirowski B Harvey SJ: DDR1-deficient mice show localized subepithelial GBM thickening with focal loss of slit diaphragms and proteinuria. *Kidney Int.* 2004;66(1):102–11. 10.1111/j.1523-1755.2004.00712.x 15200417

[180] Okamoto K Tokunaga K Doi K: Common variation in *GPC5* is associated with acquired nephrotic syndrome. *Nat Genet.* 2011;43(5):459–63. 10.1038/ng.792 21441931

[181] Dai C Stolz DB Bastacky SI: Essential role of integrin-linked kinase in podocyte biology: Bridging the integrin and slit diaphragm signaling. *J Am Soc Nephrol.* 2006;17(8):2164–75. 10.1681/ASN.2006010033 16837631

[182] El-Aouni C Herbach N Blattner SM: Podocyte-specific deletion of integrin-linked kinase results in severe glomerular basement membrane alterations and progressive glomerulosclerosis. *J Am Soc Nephrol.* 2006;17(5):1334–44. 10.1681/ASN.2005090921 16611717

[183] Noakes PG Miner JH Gautam M: The renal glomerulus of mice lacking s-laminin/laminin beta 2: nephrosis despite molecular compensation by laminin beta 1. *Nat Genet.* 1995;10(4):400–6. 10.1038/ng0895-400 7670489

[184] Jarad G Cunningham J Shaw AS: Proteinuria precedes podocyte abnormalities in *Lamb2* *^-/-^* mice, implicating the glomerular basement membrane as an albumin barrier. *J Clin Invest.* 2006;116(8):2272–9. 10.1172/JCI28414 16886065 PMC1523402

[185] Chen YM Kikkawa Y Miner JH: A missense *LAMB2* mutation causes congenital nephrotic syndrome by impairing laminin secretion. *J Am Soc Nephrol.* 2011;22(5):849–58. 10.1681/ASN.2010060632 21511833 PMC3083307

[186] Chen YM Zhou Y Go G: *Laminin* *β2* gene missense mutation produces endoplasmic reticulum stress in podocytes. *J Am Soc Nephrol.* 2013;24(8):1223–33. 10.1681/ASN.2012121149 23723427 PMC3736718

[187] Sugar T Wassenhove-McCarthy DJ Esko JD: Podocyte-specific deletion of NDST1, a key enzyme in the sulfation of heparan sulfate glycosaminoglycans, leads to abnormalities in podocyte organization *in vivo*. *Kidney Int.* 2014;85(2):307–18. 10.1038/ki.2013.281 23924956 PMC4624314

[188] Potla U Ni J Vadaparampil J: Podocyte-specific RAP1GAP expression contributes to focal segmental glomerulosclerosis-associated glomerular injury. *J Clin Invest.* 2014;124(4):1757–69. 10.1172/JCI67846 24642466 PMC3973092

[189] Tian X Kim JJ Monkley SM: Podocyte-associated talin1 is critical for glomerular filtration barrier maintenance. *J Clin Invest.* 2014;124(3):1098–113. 10.1172/JCI69778 24531545 PMC3934159

[190] Berkovic SF Dibbens LM Oshlack A: Array-based gene discovery with three unrelated subjects shows SCARB2/LIMP-2 deficiency causes myoclonus epilepsy and glomerulosclerosis. *Am J Hum Genet.* 2008;82(3):673–84. 10.1016/j.ajhg.2007.12.019 18308289 PMC2427287

[191] Diomedi-Camassei F Di Giandomenico S Santorelli FM: *COQ2* nephropathy: a newly described inherited mitochondriopathy with primary renal involvement. *J Am Soc Nephrol.* 2007;18(10):2773–80. 10.1681/ASN.2006080833 17855635

[192] Heeringa SF Chernin G Chaki M: *COQ6* mutations in human patients produce nephrotic syndrome with sensorineural deafness. *J Clin Invest.* 2011;121(5):2013–24. 10.1172/JCI45693 21540551 PMC3083770

[193] Casalena G Krick S Daehn I: Mpv17 in mitochondria protects podocytes against mitochondrial dysfunction and apoptosis *in vivo* and *in vitro*. *Am J Physiol Renal Physiol.* 2014;306(11):F1372–80. 10.1152/ajprenal.00608.2013 24598802 PMC4042102

[194] Löwik MM Hol FA Steenbergen EJ: Mitochondrial tRNA ^Leu(UUR)^ mutation in a patient with steroid-resistant nephrotic syndrome and focal segmental glomerulosclerosis. *Nephrol Dial Transplant.* 2005;20(2):336–41. 10.1093/ndt/gfh546 15585516

[195] Ising C Koehler S Brähler S: Inhibition of insulin/IGF-1 receptor signaling protects from mitochondria-mediated kidney failure. *EMBO Mol Med.* 2015;7(3):275–87. 10.15252/emmm.201404916 25643582 PMC4364945

[196] Hartleben B Gödel M Meyer-Schwesinger C: Autophagy influences glomerular disease susceptibility and maintains podocyte homeostasis in aging mice. *J Clin Invest.* 2010;120(4):1084–96. 10.1172/JCI39492 20200449 PMC2846040

[197] Cinà DP Onay T Paltoo A: Inhibition of MTOR disrupts autophagic flux in podocytes. *J Am Soc Nephrol.* 2012;23(3):412–20. 10.1681/ASN.2011070690 22193387 PMC3294311

[198] Riediger F Quack I Qadri F: Prorenin receptor is essential for podocyte autophagy and survival. *J Am Soc Nephrol.* 2011;22(12):2193–202. 10.1681/ASN.2011020200 22034640 PMC3279931

[199] Oshima Y Kinouchi K Ichihara A: Prorenin receptor is essential for normal podocyte structure and function. *J Am Soc Nephrol.* 2011;22(12):2203–12. 10.1681/ASN.2011020202 22052048 PMC3279932

[200] Li C Siragy HM: (Pro)renin receptor regulates autophagy and apoptosis in podocytes exposed to high glucose. *Am J Physiol Endocrinol Metab.* 2015;309(3):E302–10. 10.1152/ajpendo.00603.2014 26081285 PMC4525115

[201] Chen J Chen MX Fogo AB: mVps34 deletion in podocytes causes glomerulosclerosis by disrupting intracellular vesicle trafficking. *J Am Soc Nephrol.* 2013;24(2):198–207. 10.1681/ASN.2012010101 23291473 PMC3559479

[202] Bechtel W Helmstädter M Balica J: Vps34 deficiency reveals the importance of endocytosis for podocyte homeostasis. *J Am Soc Nephrol.* 2013;24(5):727–43. 10.1681/ASN.2012070700 23492732 PMC3636793

[203] Weins A Wong JS Basgen JM: Dendrin ablation prolongs life span by delaying kidney failure. *Am J Pathol.* 2015;185(8):2143–57. 10.1016/j.ajpath.2015.04.011 26073036 PMC4530132

[204] Li X Zhang X Li X: The role of survivin in podocyte injury induced by puromycin aminonucleoside. *Int J Mol Sci.* 2014;15(4):6657–73. 10.3390/ijms15046657 24747598 PMC4013653

[205] Campbell KN Wong JS Gupta R: Yes-associated protein (YAP) promotes cell survival by inhibiting proapoptotic dendrin signaling. *J Biol Chem.* 2013;288(24):17057–62. 10.1074/jbc.C113.457390 23667252 PMC3682510

[206] Schwartzman M Reginensi A Wong JS: Podocyte-Specific Deletion of Yes-Associated Protein Causes FSGS and Progressive Renal Failure. *J Am Soc Nephrol.* 2016;27(1):216–26. 10.1681/ASN.2014090916 26015453 PMC4696566

[207] Eremina V Sood M Haigh J: Glomerular-specific alterations of VEGF-A expression lead to distinct congenital and acquired renal diseases. *J Clin Invest.* 2003;111(5):707–16. 10.1172/JCI17423 12618525 PMC151905

[208] Eremina V Cui S Gerber H: Vascular endothelial growth factor a signaling in the podocyte-endothelial compartment is required for mesangial cell migration and survival. *J Am Soc Nephrol.* 2006;17(3):724–35. 10.1681/ASN.2005080810 16436493

[209] Kato H Gruenwald A Suh JH: Wnt/β-catenin pathway in podocytes integrates cell adhesion, differentiation, and survival. *J Biol Chem.* 2011;286(29):26003–15. 10.1074/jbc.M111.223164 21613219 PMC3138306

[210] Niranjan T Bielesz B Gruenwald A: The Notch pathway in podocytes plays a role in the development of glomerular disease. *Nat Med.* 2008;14(3):290–8. 10.1038/nm1731 18311147

[211] Kretzler M Teixeira VP Unschuld PG: Integrin-linked kinase as a candidate downstream effector in proteinuria. *FASEB J.* 2001;15(10):1843–5. 10.1096/fj.00-0832fje 11481249

[212] Teixeira Vde P Blattner SM Li M: Functional consequences of integrin-linked kinase activation in podocyte damage. *Kidney Int.* 2005;67(2):514–23. 10.1111/j.1523-1755.2005.67108.x 15673299

[213] Kang YS Li Y Dai C: Inhibition of integrin-linked kinase blocks podocyte epithelial-mesenchymal transition and ameliorates proteinuria. *Kidney Int.* 2010;78(4):363–73. 10.1038/ki.2010.137 20505657 PMC3065782

[214] He JC Husain M Sunamoto M: Nef stimulates proliferation of glomerular podocytes through activation of Src-dependent Stat3 and MAPK1,2 pathways. *J Clin Invest.* 2004;114(5):643–51. 10.1172/JCI21004 15343382 PMC514582

[215] Zuo Y Matsusaka T Zhong J: HIV-1 genes *vpr* and *nef* synergistically damage podocytes, leading to glomerulosclerosis. *J Am Soc Nephrol.* 2006;17(10):2832–43. 10.1681/ASN.2005080878 16988066

[216] Waters AM Wu MY Onay T: Ectopic notch activation in developing podocytes causes glomerulosclerosis. *J Am Soc Nephrol.* 2008;19(6):1139–57. 10.1681/ASN.2007050596 18337488 PMC2396929

[217] Wasik AA Polianskyte-Prause Z Dong MQ: Septin 7 forms a complex with CD2AP and nephrin and regulates glucose transporter trafficking. *Mol Biol Cell.* 2012;23(17):3370–9. 10.1091/mbc.E11-12-1010 22809625 PMC3431928

[218] Schiffer M Bitzer M Roberts IS: Apoptosis in podocytes induced by TGF-beta and Smad7. *J Clin Invest.* 2001;108(8):807–16. 10.1172/JCI12367 11560950 PMC200928

[219] Inoki K Mori H Wang J: mTORC1 activation in podocytes is a critical step in the development of diabetic nephropathy in mice. *J Clin Invest.* 2011;121(6):2181–96. 10.1172/JCI44771 21606597 PMC3104745

[220] Dai C Stolz DB Kiss LP: Wnt/beta-catenin signaling promotes podocyte dysfunction and albuminuria. *J Am Soc Nephrol.* 2009;20(9):1997–2008. 10.1681/ASN.2009010019 19628668 PMC2736766

[221] Canaud G Bienaimé F Viau A: AKT2 is essential to maintain podocyte viability and function during chronic kidney disease. *Nat Med.* 2013;19(10):1288–96. 10.1038/nm.3313 24056770

[222] Yang Y Guo L Blattner SM: Formation and phosphorylation of the PINCH-1-integrin linked kinase-alpha-parvin complex are important for regulation of renal glomerular podocyte adhesion, architecture, and survival. *J Am Soc Nephrol.* 2005;16(7):1966–76. 10.1681/ASN.2004121112 15872073

[223] Rüster C Franke S Wenzel U: Podocytes of AT2 receptor knockout mice are protected from angiotensin II-mediated RAGE induction. *Am J Nephrol.* 2011;34(4):309–17. 10.1159/000329321 21846974

[224] Heikkilä E Juhila J Lassila M: beta-Catenin mediates adriamycin-induced albuminuria and podocyte injury in adult mouse kidneys. *Nephrol Dial Transplant.* 2010;25(8):2437–46. 10.1093/ndt/gfq076 20237062

[225] Jiang L Xu L Song Y: Calmodulin-dependent protein kinase II/cAMP response element-binding protein/Wnt/β-catenin signaling cascade regulates angiotensin II-induced podocyte injury and albuminuria. *J Biol Chem.* 2013;288(32):23368–79. 10.1074/jbc.M113.460394 23803607 PMC3743506

[226] Lu T He JC Wang ZH: HIV-1 Nef disrupts the podocyte actin cytoskeleton by interacting with diaphanous interacting protein. *J Biol Chem.* 2008;283(13):8173–82. 10.1074/jbc.M708920200 18234668 PMC2276381

[227] Welsh GI Hale LJ Eremina V: Insulin signaling to the glomerular podocyte is critical for normal kidney function. *Cell Metab.* 2010;12(4):329–40. 10.1016/j.cmet.2010.08.015 20889126 PMC4949331

[228] Hale LJ Welsh GI Perks CM: Insulin-like growth factor-II is produced by, signals to and is an important survival factor for the mature podocyte in man and mouse. *J Pathol.* 2013;230(1):95–106. 10.1002/path.4165 23299523

[229] Hale LJ Hurcombe J Lay A: Insulin directly stimulates VEGF-A production in the glomerular podocyte. *Am J Physiol Renal Physiol.* 2013;305(2):F182–8. 10.1152/ajprenal.00548.2012 23698113 PMC3725664

[230] Brähler S Ising C Hagmann H: Intrinsic proinflammatory signaling in podocytes contributes to podocyte damage and prolonged proteinuria. *Am J Physiol Renal Physiol.* 2012;303(10):F1473–85. 10.1152/ajprenal.00031.2012 22975019

[231] Brähler S Ising C Barrera Aranda B: The NF-κB essential modulator (NEMO) controls podocyte cytoskeletal dynamics independently of NF-κB. *Am J Physiol Renal Physiol.* 2015;309(7):F617–26. 10.1152/ajprenal.00059.2015 26268269

[232] Lin CL Wang FS Hsu YC: Modulation of notch-1 signaling alleviates vascular endothelial growth factor-mediated diabetic nephropathy. *Diabetes.* 2010;59(8):1915–25. 10.2337/db09-0663 20522599 PMC2911050

[233] El Machhour F Keuylian Z Kavvadas P: Activation of Notch3 in Glomeruli Promotes the Development of Rapidly Progressive Renal Disease. *J Am Soc Nephrol.* 2015;26(7):1561–75. 10.1681/ASN.2013090968 25421557 PMC4483574

[234] Saurus P Kuusela S Lehtonen E: Podocyte apoptosis is prevented by blocking the Toll-like receptor pathway. *Cell Death Dis.* 2015;6:e1752. 10.1038/cddis.2015.125 25950482 PMC4669704

[235] Gödel M Hartleben B Herbach N: Role of mTOR in podocyte function and diabetic nephropathy in humans and mice. *J Clin Invest.* 2011;121(6):2197–209. 10.1172/JCI44774 21606591 PMC3104746

[236] Hyvönen ME Saurus P Wasik A: Lipid phosphatase SHIP2 downregulates insulin signalling in podocytes. *Mol Cell Endocrinol.* 2010;328(1–2):70–9. 10.1016/j.mce.2010.07.016 20654688

[237] Das R Xu S Quan X: Upregulation of mitochondrial Nox4 mediates TGF-β-induced apoptosis in cultured mouse podocytes. *Am J Physiol Renal Physiol.* 2014;306(2):F155–67. 10.1152/ajprenal.00438.2013 24259511

[238] Feng X Lu TC Chuang PY: Reduction of Stat3 activity attenuates HIV-induced kidney injury. *J Am Soc Nephrol.* 2009;20(10):2138–46. 10.1681/ASN.2008080879 19608706 PMC2754106

[239] Dai Y Gu L Yuan W: Podocyte-specific deletion of signal transducer and activator of transcription 3 attenuates nephrotoxic serum-induced glomerulonephritis. *Kidney Int.* 2013;84(5):950–61. 10.1038/ki.2013.197 23842188 PMC3797218

[240] Abkhezr M Dryer SE: STAT3 regulates steady-state expression of synaptopodin in cultured mouse podocytes. *Mol Pharmacol.* 2015;87(2):231–9. 10.1124/mol.114.094508 25425624

[241] Chugh SS: Transcriptional regulation of podocyte disease. *Transl Res.* 2007;149(5):237–42. 10.1016/j.trsl.2007.01.002 17466922 PMC1974875

[242] Zhang SY Kamal M Dahan K: c-mip impairs podocyte proximal signaling and induces heavy proteinuria. *Sci Signal.* 2010;3(122):ra39. 10.1126/scisignal.2000678 20484117 PMC2897853

[243] Sendeyo K Audard V Zhang SY: Upregulation of c-mip is closely related to podocyte dysfunction in membranous nephropathy. *Kidney Int.* 2013;83(3):414–25. 10.1038/ki.2012.426 23302718 PMC3927461

[244] Takemoto M He L Norlin J: Large-scale identification of genes implicated in kidney glomerulus development and function. *EMBO J.* 2006;25(5):1160–74. 10.1038/sj.emboj.7601014 16498405 PMC1409724

[245] Brukamp K Jim B Moeller MJ: Hypoxia and podocyte-specific *Vhlh* deletion confer risk of glomerular disease. *Am J Physiol Renal Physiol.* 2007;293(4):F1397–407. 10.1152/ajprenal.00133.2007 17609290

[246] Steenhard BM Isom K Stroganova L: Deletion of von Hippel-Lindau in glomerular podocytes results in glomerular basement membrane thickening, ectopic subepithelial deposition of collagen {alpha}1{alpha}2{alpha}1(IV), expression of neuroglobin, and proteinuria. *Am J Pathol.* 2010;177(1):84–96. 10.2353/ajpath.2010.090767 20522651 PMC2893653

[247] Ding M Cui S Li C: Loss of the tumor suppressor Vhlh leads to upregulation of Cxcr4 and rapidly progressive glomerulonephritis in mice. *Nat Med.* 2006;12(9):1081–7. 10.1038/nm1460 16906157

[248] Mallipattu SK Horne SJ D'Agati V: Krüppel-like factor 6 regulates mitochondrial function in the kidney. *J Clin Invest.* 2015;125(3):1347–61. 10.1172/JCI77084 25689250 PMC4362257

[249] Chen H Lun Y Ovchinnikov D: Limb and kidney defects in *Lmx1b* mutant mice suggest an involvement of *LMX1B* in human nail patella syndrome. *Nat Genet.* 1998;19(1):51–5. 10.1038/ng0598-51 9590288

[250] Morello R Zhou G Dreyer SD: Regulation of glomerular basement membrane collagen expression by *LMX1B* contributes to renal disease in nail patella syndrome. *Nat Genet.* 2001;27(2):205–8. 10.1038/84853 11175791

[251] Miner JH Morello R Andrews KL: Transcriptional induction of slit diaphragm genes by Lmx1b is required in podocyte differentiation. *J Clin Invest.* 2002;109(8):1065–72. 10.1172/JCI13954 11956244 PMC150942

[252] Rohr C Prestel J Heidet L: The LIM-homeodomain transcription factor Lmx1b plays a crucial role in podocytes. *J Clin Invest.* 2002;109(8):1073–82. 10.1172/JCI13961 11956245 PMC150943

[253] Suleiman H Heudobler D Raschta AS: The podocyte-specific inactivation of *Lmx1b*, *Ldb1* and *E2a* yields new insight into a transcriptional network in podocytes. *Dev Biol.* 2007;304(2):701–12. 10.1016/j.ydbio.2007.01.020 17316599

[254] Burghardt T Kastner J Suleiman H: LMX1B is essential for the maintenance of differentiated podocytes in adult kidneys. *J Am Soc Nephrol.* 2013;24(11):1830–48. 10.1681/ASN.2012080788 23990680 PMC3810075

[255] Sadl V Jin F Yu J: The mouse *Kreisler* ( *Krml1/MafB*) segmentation gene is required for differentiation of glomerular visceral epithelial cells. *Dev Biol.* 2002;249(1):16–29. 10.1006/dbio.2002.0751 12217315

[256] Moriguchi T Hamada M Morito N: MafB is essential for renal development and F4/80 expression in macrophages. *Mol Cell Biol.* 2006;26(15):5715–27. 10.1128/MCB.00001-06 16847325 PMC1592773

[257] Wang Y Jarad G Tripathi P: Activation of NFAT signaling in podocytes causes glomerulosclerosis. *J Am Soc Nephrol.* 2010;21(10):1657–66. 10.1681/ASN.2009121253 20651158 PMC3013542

[258] Nijenhuis T Sloan AJ Hoenderop JG: Angiotensin II contributes to podocyte injury by increasing TRPC6 expression via an NFAT-mediated positive feedback signaling pathway. *Am J Pathol.* 2011;179(4):1719–32. 10.1016/j.ajpath.2011.06.033 21839714 PMC3181349

[259] Barua M Stellacci E Stella L: Mutations in *PAX2* associate with adult-onset FSGS. *J Am Soc Nephrol.* 2014;25(9):1942–53. 10.1681/ASN.2013070686 24676634 PMC4147972

[260] Cui S Li C Ema M: Rapid isolation of glomeruli coupled with gene expression profiling identifies downstream targets in Pod1 knockout mice. *J Am Soc Nephrol.* 2005;16(11):3247–55. 10.1681/ASN.2005030278 16207825

[261] Zhou Y Kong X Zhao P: Peroxisome proliferator-activated receptor-α is renoprotective in doxorubicin-induced glomerular injury. *Kidney Int.* 2011;79(12):1302–11. 10.1038/ki.2011.17 21368746

[262] Matsui I Ito T Kurihara H: Snail, a transcriptional regulator, represses nephrin expression in glomerular epithelial cells of nephrotic rats. *Lab Invest.* 2007;87(3):273–83. 10.1038/labinvest.3700518 17260001

[263] Buelli S Rosanò L Gagliardini E: *β*-arrestin-1 drives endothelin-1-mediated podocyte activation and sustains renal injury. *J Am Soc Nephrol.* 2014;25(3):523–33. 10.1681/ASN.2013040362 24371298 PMC3935587

[264] Ito S Ikeda M Takata A: Nephrotic syndrome and end-stage renal disease with *WT1* mutation detected at 3 years. *Pediatr Nephrol.* 1999;13(9):790–1. 10.1007/s004670050702 10603123

[265] Guo JK Menke AL Gubler MC: WT1 is a key regulator of podocyte function: reduced expression levels cause crescentic glomerulonephritis and mesangial sclerosis. *Hum Mol Genet.* 2002;11(6):651–9. 10.1093/hmg/11.6.651 11912180

[266] Patek CE Fleming S Miles CG: Murine Denys-Drash syndrome: evidence of podocyte de-differentiation and systemic mediation of glomerulosclerosis. *Hum Mol Genet.* 2003;12(18):2379–94. 10.1093/hmg/ddg240 12915483

[267] Chau YY Brownstein D Mjoseng H: Acute multiple organ failure in adult mice deleted for the developmental regulator Wt1. *PLoS Genet.* 2011;7(12):e1002404. 10.1371/journal.pgen.1002404 22216009 PMC3245305

[268] Hall G Gbadegesin RA Lavin P: A novel missense mutation of Wilms' Tumor 1 causes autosomal dominant FSGS. *J Am Soc Nephrol.* 2015;26(4):831–43. 10.1681/ASN.2013101053 25145932 PMC4378093

[269] Kumar PA Kotlyarevska K Dejkhmaron P: Growth hormone (GH)-dependent expression of a natural antisense transcript induces zinc finger E-box-binding homeobox 2 (ZEB2) in the glomerular podocyte: a novel action of gh with implications for the pathogenesis of diabetic nephropathy. *J Biol Chem.* 2010;285(41):31148–56. 10.1074/jbc.M110.132332 20682777 PMC2951188

[270] Liu G Clement LC Kanwar YS: ZHX proteins regulate podocyte gene expression during the development of nephrotic syndrome. *J Biol Chem.* 2006;281(51):39681–92. 10.1074/jbc.M606664200 17056598

[271] Lin Y Rao J Zha XL: Angiopoietin-like 3 induces podocyte F-actin rearrangement through integrin α(V)β₃/FAK/PI3K pathway-mediated Rac1 activation. *Biomed Res Int.* 2013;2013: 135608. 10.1155/2013/135608 24294595 PMC3835706

[272] Fiorina P Vergani A Bassi R: Role of podocyte B7-1 in diabetic nephropathy. *J Am Soc Nephrol.* 2014;25(7):1415–29. 10.1681/ASN.2013050518 24676639 PMC4073425

[273] Iglesias-de la Cruz MC Ziyadeh FN Isono M: Effects of high glucose and TGF-beta1 on the expression of collagen IV and vascular endothelial growth factor in mouse podocytes. *Kidney Int.* 2002;62(3):901–13. 10.1046/j.1523-1755.2002.00528.x 12164872

[274] Han SY Kang YS Jee YH: High glucose and angiotensin II increase beta1 integrin and integrin-linked kinase synthesis in cultured mouse podocytes. *Cell Tissue Res.* 2006;323(2):321–32. 10.1007/s00441-005-0065-4 16189717

[275] Ha TS: High glucose and advanced glycosylated end-products affect the expression of alpha-actinin-4 in glomerular epithelial cells. *Nephrology (Carlton).* 2006;11(5):435–41. 10.1111/j.1440-1797.2006.00668.x 17014558

[276] Kim NH Rincon-Choles H Bhandari B: Redox dependence of glomerular epithelial cell hypertrophy in response to glucose. *Am J Physiol Renal Physiol.* 2006;290(3):F741–51. 10.1152/ajprenal.00313.2005 16234311

[277] De Petris L Hruska KA Chiechio S: Bone morphogenetic protein-7 delays podocyte injury due to high glucose. *Nephrol Dial Transplant.* 2007;22(12):3442–50. 10.1093/ndt/gfm503 17686813

[278] Wei C El Hindi S Li J: Circulating urokinase receptor as a cause of focal segmental glomerulosclerosis. *Nat Med.* 2011;17(8):952–60. 10.1038/nm.2411 21804539 PMC4089394

[279] Koukouritaki SB Vardaki EA Papakonstanti EA: TNF-alpha induces actin cytoskeleton reorganization in glomerular epithelial cells involving tyrosine phosphorylation of paxillin and focal adhesion kinase. *Mol Med.* 1999;5(6):382–92. 10415163 PMC2230436

[280] Saito Y Okamura M Nakajima S: Suppression of nephrin expression by TNF-alpha via interfering with the cAMP-retinoic acid receptor pathway. *Am J Physiol Renal Physiol.* 2010;298(6):F1436–44. 10.1152/ajprenal.00512.2009 20237236

[281] Bitzan M Babayeva S Vasudevan A: TNFα pathway blockade ameliorates toxic effects of FSGS plasma on podocyte cytoskeleton and β3 integrin activation. *Pediatr Nephrol.* 2012;27(12):2217–26. 10.1007/s00467-012-2163-3 22538781

[282] Tian D Jacobo SM Billing D: Antagonistic regulation of actin dynamics and cell motility by TRPC5 and TRPC6 channels. *Sci Signal.* 2010;3(145):ra77. 10.1126/scisignal.2001200 20978238 PMC3071756

[283] Yoshida S Nagase M Shibata S: Podocyte injury induced by albumin overload *in vivo* and *in vitro*: involvement of TGF-beta and p38 MAPK. *Nephron Exp Nephrol.* 2008;108(3):e57–68. 10.1159/000124236 18391505

[284] He F Chen S Wang H: Regulation of CD2-associated protein influences podocyte endoplasmic reticulum stress-mediated apoptosis induced by albumin overload. *Gene.* 2011;484(1–2):18–25. 10.1016/j.gene.2011.05.025 21679752

[285] Okamura K Dummer P Kopp J: Endocytosis of albumin by podocytes elicits an inflammatory response and induces apoptotic cell death. *PLoS One.* 2013;8(1):e54817. 10.1371/journal.pone.0054817 23382978 PMC3557279

[286] Shibata S Nagase M Yoshida S: Podocyte as the target for aldosterone: roles of oxidative stress and Sgk1. *Hypertension.* 2007;49(2):355–64. 10.1161/01.HYP.0000255636.11931.a2 17200434

[287] Zhu C Huang S Yuan Y: Mitochondrial dysfunction mediates aldosterone-induced podocyte damage: a therapeutic target of PPARγ. *Am J Pathol.* 2011;178(5):2020–31. 10.1016/j.ajpath.2011.01.029 21514419 PMC3081205

[288] Su M Dhoopun AR Yuan Y: Mitochondrial dysfunction is an early event in aldosterone-induced podocyte injury. *Am J Physiol Renal Physiol.* 2013;305(4):F520–31. 10.1152/ajprenal.00570.2012 23761667

[289] Dai R Lin Y Liu H: A vital role for Angptl3 in the PAN-induced podocyte loss by affecting detachment and apoptosis *in vitro*. *BMC Nephrol.* 2015;16:38. 10.1186/s12882-015-0034-4 25884163 PMC4383073

[290] Ding G Reddy K Kapasi AA: Angiotensin II induces apoptosis in rat glomerular epithelial cells. *Am J Physiol Renal Physiol.* 2002;283(1):F173–80. 10.1152/ajprenal.00240.2001 12060599

[291] Jia J Ding G Zhu J: Angiotensin II infusion induces nephrin expression changes and podocyte apoptosis. *Am J Nephrol.* 2008;28(3):500–7. 10.1159/000113538 18204248 PMC2630486

[292] Zhang H Ding J Fan Q: TRPC6 up-regulation in Ang II-induced podocyte apoptosis might result from ERK activation and NF-kappaB translocation. *Exp Biol Med (Maywood).* 2009;234(9):1029–36. 10.3181/0901-RM-11 19546355

[293] Ren Z Liang W Chen C: Angiotensin II induces nephrin dephosphorylation and podocyte injury: role of caveolin-1. *Cell Signal.* 2012;24(2):443–50. 10.1016/j.cellsig.2011.09.022 21982880 PMC3237911

[294] Sanchez-Niño MD Sanz AB Sanchez-Lopez E: HSP27/HSPB1 as an adaptive podocyte antiapoptotic protein activated by high glucose and angiotensin II. *Lab Invest.* 2012;92(1):32–45. 10.1038/labinvest.2011.138 21931298

[295] Sieber J Lindenmeyer MT Kampe K: Regulation of podocyte survival and endoplasmic reticulum stress by fatty acids. *Am J Physiol Renal Physiol.* 2010;299(4):F821–9. 10.1152/ajprenal.00196.2010 20668104 PMC2957252

[296] Sieber J Weins A Kampe K: Susceptibility of podocytes to palmitic acid is regulated by stearoyl-CoA desaturases 1 and 2. *Am J Pathol.* 2013;183(3):735–44. 10.1016/j.ajpath.2013.05.023 23867797 PMC3763774

[297] Kampe K Sieber J Orellana JM: Susceptibility of podocytes to palmitic acid is regulated by fatty acid oxidation and inversely depends on acetyl-CoA carboxylases 1 and 2. *Am J Physiol Renal Physiol.* 2014;306(4):F401–9. 10.1152/ajprenal.00454.2013 24338821 PMC3920022

[298] Susztak K Raff AC Schiffer M: Glucose-induced reactive oxygen species cause apoptosis of podocytes and podocyte depletion at the onset of diabetic nephropathy. *Diabetes.* 2006;55(1):225–33. 10.2337/diabetes.55.01.06.db05-0894 16380497

[299] Peters I Tossidou I Achenbach J: IGF-binding protein-3 modulates TGF-beta/BMP-signaling in glomerular podocytes. *J Am Soc Nephrol.* 2006;17(6):1644–56. 10.1681/ASN.2005111209 16672319

[300] Bussolati B Deregibus MC Fonsato V: Statins prevent oxidized LDL-induced injury of glomerular podocytes by activating the phosphatidylinositol 3-kinase/AKT-signaling pathway. *J Am Soc Nephrol.* 2005;16(7):1936–47. 10.1681/ASN.2004080629 15843472

[301] Schiffer M Mundel P Shaw AS: A novel role for the adaptor molecule CD2-associated protein in transforming growth factor-beta-induced apoptosis. *J Biol Chem.* 2004;279(35):37004–12. 10.1074/jbc.M403534200 15213232

[302] Wada T Pippin JW Terada Y: The cyclin-dependent kinase inhibitor p21 is required for TGF-beta1-induced podocyte apoptosis. *Kidney Int.* 2005;68(4):1618–29. 10.1111/j.1523-1755.2005.00574.x 16164639

[303] Wu DT Bitzer M Ju W: TGF-beta concentration specifies differential signaling profiles of growth arrest/differentiation and apoptosis in podocytes. *J Am Soc Nephrol.* 2005;16(11):3211–21. 10.1681/ASN.2004121055 16207831

[304] Jung KY Chen K Kretzler M: TGF-beta1 regulates the PINCH-1-integrin-linked kinase-alpha-parvin complex in glomerular cells. *J Am Soc Nephrol.* 2007;18(1):66–73. 10.1681/ASN.2006050421 17167118

[305] Yadav A Vallabu S Arora S: ANG II promotes autophagy in podocytes. *Am J Physiol Cell Physiol.* 2010;299(2):C488–96. 10.1152/ajpcell.00424.2009 20484657 PMC2928643

[306] Ma T Zhu J Chen X: High glucose induces autophagy in podocytes. *Exp Cell Res.* 2013;319(6):779–89. 10.1016/j.yexcr.2013.01.018 23384600 PMC3628680

[307] Liu Y: New insights into epithelial-mesenchymal transition in kidney fibrosis. *J Am Soc Nephrol.* 2010;21(2):212–22. 10.1681/ASN.2008121226 20019167 PMC4554339

[308] Li Y Kang YS Dai C: Epithelial-to-mesenchymal transition is a potential pathway leading to podocyte dysfunction and proteinuria. *Am J Pathol.* 2008;172(2):299–308. 10.2353/ajpath.2008.070057 18202193 PMC2312375

[309] Husain M Gusella GL Klotman ME: HIV-1 Nef induces proliferation and anchorage-independent growth in podocytes. *J Am Soc Nephrol.* 2002;13(7):1806–15. 10.1097/01.ASN.0000019642.55998.69 12089376

[310] Sunamoto M Husain M He JC: Critical role for Nef in HIV-1-induced podocyte dedifferentiation. *Kidney Int.* 2003;64(5):1695–701. 10.1046/j.1523-1755.2003.00283.x 14531802

[311] Bruggeman LA Drawz PE Kahoud N: TNFR2 interposes the proliferative and NF-κB-mediated inflammatory response by podocytes to TNF-α. *Lab Invest.* 2011;91(3):413–25. 10.1038/labinvest.2010.199 21221075 PMC3075956

[312] Clement LC Macé C Avila-Casado C: Circulating angiopoietin-like 4 links proteinuria with hypertriglyceridemia in nephrotic syndrome. *Nat Med.* 2014;20(1):37–46. 10.1038/nm.3396 24317117 PMC4114723

[313] Isermann B Vinnikov IA Madhusudhan T: Activated protein C protects against diabetic nephropathy by inhibiting endothelial and podocyte apoptosis. *Nat Med.* 2007;13(11):1349–58. 10.1038/nm1667 17982464

[314] Madhusudhan T Wang H Straub BK: Cytoprotective signaling by activated protein C requires protease-activated receptor-3 in podocytes. *Blood.* 2012;119(3):874–83. 10.1182/blood-2011-07-365973 22117049 PMC3398751

[315] Mitu GM Wang S Hirschberg R: BMP7 is a podocyte survival factor and rescues podocytes from diabetic injury. *Am J Physiol Renal Physiol.* 2007;293(5):F1641–8. 10.1152/ajprenal.00179.2007 17804487

[316] Foster RR Hole R Anderson K: Functional evidence that vascular endothelial growth factor may act as an autocrine factor on human podocytes. *Am J Physiol Renal Physiol.* 2003;284(6):F1263–73. 10.1152/ajprenal.00276.2002 12620928

[317] Foster RR Saleem MA Mathieson PW: Vascular endothelial growth factor and nephrin interact and reduce apoptosis in human podocytes. *Am J Physiol Renal Physiol.* 2005;288(1):F48–57. 10.1152/ajprenal.00146.2004 15339792

[318] Müller-Deile J Worthmann K Saleem M: The balance of autocrine VEGF-A and VEGF-C determines podocyte survival. *Am J Physiol Renal Physiol.* 2009;297(6):F1656–67. 10.1152/ajprenal.00275.2009 19828679

[319] Foster RR Satchell SC Seckley J: VEGF-C promotes survival in podocytes. *Am J Physiol Renal Physiol.* 2006;291(1):F196–207. 10.1152/ajprenal.00431.2005 16525158

[320] Barisoni L Schnaper HW Kopp JB: A proposed taxonomy for the podocytopathies: a reassessment of the primary nephrotic diseases. *Clin J Am Soc Nephrol.* 2007;2(3):529–42. 10.2215/CJN.04121206 17699461

[321] Chiang C Inagi R: Glomerular diseases: genetic causes and future therapeutics. *Nat Rev Nephrol.* 2010;6(9):539–54. 10.1038/nrneph.2010.103 20644582

[322] Smeets B Moeller MJ: Parietal epithelial cells and podocytes in glomerular diseases. *Semin Nephrol.* 2012;32(4):357–67. 10.1016/j.semnephrol.2012.06.007 22958490

[323] Pollak MR: The genetic basis of FSGS and steroid-resistant nephrosis. *Semin Nephrol.* 2003;23(2):141–6. 10.1053/snep.2003.50014 12704574

[324] Löwik MM Groenen PJ Levtchenko EN: Molecular genetic analysis of podocyte genes in focal segmental glomerulosclerosis--a review. *Eur J Pediatr.* 2009;168(11):1291–304. 10.1007/s00431-009-1017-x 19562370 PMC2745545

[325] Kuppe C Gröne HJ Ostendorf T: Common histological patterns in glomerular epithelial cells in secondary focal segmental glomerulosclerosis. *Kidney Int.* 2015;88(5):990–8. 10.1038/ki.2015.116 25853334

[326] Wiggins RC: The spectrum of podocytopathies: a unifying view of glomerular diseases. *Kidney Int.* 2007;71(12):1205–14. 10.1038/sj.ki.5002222 17410103

[327] Fukuda A Chowdhury MA Venkatareddy MP: Growth-dependent podocyte failure causes glomerulosclerosis. *J Am Soc Nephrol.* 2012;23(8):1351–63. 10.1681/ASN.2012030271 22773827 PMC3402293

[328] Kriz W LeHir M: Pathways to nephron loss starting from glomerular diseases-insights from animal models. *Kidney Int.* 2005;67(2):404–19. 10.1111/j.1523-1755.2005.67097.x 15673288

[329] Savin VJ Sharma R Sharma M: Circulating factor associated with increased glomerular permeability to albumin in recurrent focal segmental glomerulosclerosis. *N Engl J Med.* 1996;334(14):878–83. 10.1056/NEJM199604043341402 8596570

[330] McCarthy ET Sharma M Savin VJ: Circulating permeability factors in idiopathic nephrotic syndrome and focal segmental glomerulosclerosis. *Clin J Am Soc Nephrol.* 2010;5(11):2115–21. 10.2215/CJN.03800609 20966123

[331] Bose B Cattran D: Glomerular diseases: FSGS. *Clin J Am Soc Nephrol.* 2014;9(3):626–32. 10.2215/CJN.05810513 23990165 PMC3944761

[332] Hayek SS Sever S Ko YA: Soluble Urokinase Receptor and Chronic Kidney Disease. *N Engl J Med.* 2015;373(20):1916–25. 10.1056/NEJMoa1506362 26539835 PMC4701036

[333] Regele HM Fillipovic E Langer B: Glomerular expression of dystroglycans is reduced in minimal change nephrosis but not in focal segmental glomerulosclerosis. *J Am Soc Nephrol.* 2000;11(3):403–12. 10703664 10.1681/ASN.V113403

[334] Chugh SS Clement LC Macé C: New insights into human minimal change disease: lessons from animal models. *Am J Kidney Dis.* 2012;59(2):284–92. 10.1053/j.ajkd.2011.07.024 21974967 PMC3253318

[335] Yang Y Gubler M Beaufils H: Dysregulation of podocyte phenotype in idiopathic collapsing glomerulopathy and HIV-associated nephropathy. *Nephron.* 2002;91(3):416–23. 10.1159/000064281 12119471

[336] Medapalli RK He JC Klotman PE: HIV-associated nephropathy: pathogenesis. *Curr Opin Nephrol Hypertens.* 2011;20(3):306–11. 10.1097/MNH.0b013e328345359a 21358326 PMC3153858

[337] Laurinavicius A Rennke HG: Collapsing glomerulopathy--a new pattern of renal injury. *Semin Diagn Pathol.* 2002;19(3):106–15. 12180632

[338] Barri YM Munshi NC Sukumalchantra S: Podocyte injury associated glomerulopathies induced by pamidronate. *Kidney Int.* 2004;65(2):634–41. 10.1111/j.1523-1755.2004.00426.x 14717935

[339] Albaqumi M Soos TJ Barisoni L: Collapsing glomerulopathy. *J Am Soc Nephrol.* 2006;17(10):2854–63. 10.1681/ASN.2006030225 16914539

[340] Detwiler RK Falk RJ Hogan SL: Collapsing glomerulopathy: a clinically and pathologically distinct variant of focal segmental glomerulosclerosis. *Kidney Int.* 1994;45(5):1416–24. 10.1038/ki.1994.185 8072254

[341] Atta MG Gallant JE Rahman MH: Antiretroviral therapy in the treatment of HIV-associated nephropathy. *Nephrol Dial Transplant.* 2006;21(10):2809–13. 10.1093/ndt/gfl337 16864598

[342] Gherardi D D'Agati V Chu TH: Reversal of collapsing glomerulopathy in mice with the cyclin-dependent kinase inhibitor CYC202. *J Am Soc Nephrol.* 2004;15(5):1212–22. 10.1097/01.ASN.0000124672.41036.F4 15100361

[343] Vaughan MR Pippin JW Griffin SV: ATRA induces podocyte differentiation and alters nephrin and podocin expression *in vitro* and *in vivo*. *Kidney Int.* 2005;68(1):133–44. 10.1111/j.1523-1755.2005.00387.x 15954902

[344] Albaqumi M Barisoni L: Current views on collapsing glomerulopathy. *J Am Soc Nephrol.* 2008;19(7):1276–81. 10.1681/ASN.2007080926 18287560

[345] D'Amico G: The commonest glomerulonephritis in the world: IgA nephropathy. *Q J Med.* 1987;64(245):709–27. 3329736

[346] Wyatt RJ Julian BA: IgA nephropathy. *N Engl J Med.* 2013;368(25):2402–14. 10.1056/NEJMra1206793 23782179

[347] Lemley KV Lafayette RA Safai M: Podocytopenia and disease severity in IgA nephropathy. *Kidney Int.* 2002;61(4):1475–85. 10.1046/j.1523-1755.2002.00269.x 11918755

[348] Lai KN Leung JC Chan LY: Activation of podocytes by mesangial-derived TNF-alpha: glomerulo-podocytic communication in IgA nephropathy. *Am J Physiol Renal Physiol.* 2008;294(4):F945–55. 10.1152/ajprenal.00423.2007 18256312

[349] Gagliardini E Benigni A Tomasoni S: Targeted downregulation of extracellular nephrin in human IgA nephropathy. *Am J Nephrol.* 2003;23(4):277–86. 10.1159/000072281 12853730

[350] Kramer H: Obesity and chronic kidney disease. *Contrib Nephrol.* 2006;151:1–18. 10.1159/000095315 16929130

[351] Zhou X Hurst RD Templeton D: High glucose alters actin assembly in glomerular mesangial and epithelial cells. *Lab Invest.* 1995;73(3):372–83. 7564270

[352] Chen HM Liu ZH Zeng CH: Podocyte lesions in patients with obesity-related glomerulopathy. *Am J Kidney Dis.* 2006;48(5):772–9. 10.1053/j.ajkd.2006.07.025 17059996

[353] Griffin SV Petermann AT Durvasula RV: Podocyte proliferation and differentiation in glomerular disease: role of cell-cycle regulatory proteins. *Nephrol Dial Transplant.* 2003;18(Suppl 6):vi8–13. 10.1093/ndt/gfg1069 12953035

[354] Kriz W: Podocyte is the major culprit accounting for the progression of chronic renal disease. *Microsc Res Tech.* 2002;57(4):189–95. 10.1002/jemt.10072 12012382

[355] Kikuchi M Wickman L Hodgin JB: Podometrics as a Potential Clinical Tool for Glomerular Disease Management. *Semin Nephrol.* 2015;35(3):245–55. 10.1016/j.semnephrol.2015.04.004 26215862 PMC4518207

[356] Reiser J Gupta V Kistler AD: Toward the development of podocyte-specific drugs. *Kidney Int.* 2010;77(8):662–8. 10.1038/ki.2009.559 20130528 PMC4089392

[357] Chugh SS Macé C Clement LC: Angiopoietin-like 4 based therapeutics for proteinuria and kidney disease. *Front Pharmacol.* 2014;5:23. 10.3389/fphar.2014.00023 24611049 PMC3933785

[358] Fornoni A Sageshima J Wei C: Rituximab targets podocytes in recurrent focal segmental glomerulosclerosis. *Sci Transl Med.* 2011;3(85):85ra46. 10.1126/scitranslmed.3002231 21632984 PMC3719858

[359] Yu CC Fornoni A Weins A: Abatacept in B7-1-positive proteinuric kidney disease. *N Engl J Med.* 2013;369(25):2416–23. 10.1056/NEJMoa1304572 24206430 PMC3951406

[360] Benigni A Gagliardini E Remuzzi G: Abatacept in B7-1-positive proteinuric kidney disease. *N Engl J Med.* 2014;370(13):1261–3. 10.1056/NEJMc1400502#SA1 24670179

[361] Alachkar N Carter-Monroe N Reiser J: Abatacept in B7-1-positive proteinuric kidney disease. *N Engl J Med.* 2014;370(13):1263–4. 10.1056/NEJMc1400502#SA2 24670180

[362] Salant DJ: Podocyte Expression of B7-1/CD80: Is it a Reliable Biomarker for the Treatment of Proteinuric Kidney Diseases with Abatacept? *J Am Soc Nephrol.* 2015; pii: ASN.2015080947. 10.1681/ASN.2015080947 26400567 PMC4814196

[363] Schiffer M Teng B Gu C: Pharmacological targeting of actin-dependent dynamin oligomerization ameliorates chronic kidney disease in diverse animal models. *Nat Med.* 2015;21(6):601–9. 10.1038/nm.3843 25962121 PMC4458177

[364] Lee HW Khan SQ Faridi MH: A Podocyte-Based Automated Screening Assay Identifies Protective Small Molecules. *J Am Soc Nephrol.* 2015;26(11):2741–52. 10.1681/ASN.2014090859 25858967 PMC4625676

